# GAPO: A Graph Attention-Based Reinforcement Learning Algorithm for Congestion-Aware Task Offloading in Multi-Hop Vehicular Edge Computing

**DOI:** 10.3390/s25154838

**Published:** 2025-08-06

**Authors:** Hongwei Zhao, Xuyan Li, Chengrui Li, Lu Yao

**Affiliations:** Department of Intelligent Science and Information Engineering, Shenyang University, Shenyang 110000, China

**Keywords:** edge computing, multi-hop networks, task offloading, V2X communication, graph neural network, deep reinforcement learning, attention

## Abstract

Efficient task offloading for delay-sensitive applications, such as autonomous driving, presents a significant challenge in multi-hop Vehicular Edge Computing (VEC) networks, primarily due to high vehicle mobility, dynamic network topologies, and complex end-to-end congestion problems. To address these issues, this paper proposes a graph attention-based reinforcement learning algorithm, named GAPO. The algorithm models the dynamic VEC network as an attributed graph and utilizes a graph neural network (GNN) to learn a network state representation that captures the global topological structure and node contextual information. Building on this foundation, an attention-based Actor–Critic framework makes joint offloading decisions by intelligently selecting the optimal destination and collaboratively determining the ratios for offloading and resource allocation. A multi-objective reward function, designed to minimize task latency and to alleviate link congestion, guides the entire learning process. Comprehensive simulation experiments and ablation studies show that, compared to traditional heuristic algorithms and standard deep reinforcement learning methods, GAPO significantly reduces average task completion latency and substantially decreases backbone link congestion. In conclusion, by deeply integrating the state-aware capabilities of GNNs with the decision-making abilities of DRL, GAPO provides an efficient, adaptive, and congestion-aware solution to the resource management problems in dynamic VEC environments.

## 1. Introduction

With the evolution of fifth-generation (5G) and future sixth-generation (6G) mobile communication technologies, emerging applications, such as autonomous driving, augmented reality, and smart cities, are transforming key sectors, such as transportation and communication [1,2,3]. One such application is the internet of vehicles (IoV), which demands massive data exchange and complex real-time computation between vehicles and their surroundings. However, the onboard computing units in vehicles are constrained by power consumption, cost, and physical space, making it difficult for them to independently handle these computation-intensive and delay-sensitive tasks. While traditional cloud computing solutions offer powerful computational capabilities, their centralized architecture and long communication distances result in significant transmission delays, failing to meet the stringent low-latency requirements of applications like autonomous driving. To address this challenge, Vehicular Edge Computing (VEC) has emerged. By deploying computing and storage resources at the network edge (e.g., at roadside units, RSUs), VEC brings services closer to the end-users, thereby significantly reducing latency, alleviating core network bandwidth pressure, and becoming a key technology supporting future intelligent transportation systems.

In the VEC framework, task offloading is a core function that determines whether, where, and how to migrate a vehicle’s computational tasks to edge servers for execution. However, achieving efficient task offloading in dynamic, multi-hop VEC environments faces a series of severe challenges. First, the high mobility of vehicles causes rapid changes in network topology and wireless channel conditions, making it extremely difficult to establish stable and reliable offloading links. Second, while multi-hop communication extends the range of resources available to vehicles, it also introduces more complex routing decisions and end-to-end congestion management problems. Finally, task offloading is inherently a complex multi-objective optimization problem that requires balancing multiple goals, such as minimizing task completion latency and alleviating network transmission congestion [4].

To address these challenges, researchers have proposed various technical solutions. Early work focused on traditional optimization methods and heuristic algorithms, which, despite their theoretical rigor, often struggle to adapt to the high dynamics of VEC networks. More recently, deep reinforcement learning (DRL) has become a popular approach due to its powerful decision-making capabilities. However, most existing DRL methods rely on flattened state vectors, which fail to capture the complex topological dependencies among network nodes, leading to a lack of global network awareness. While the latest research has begun to combine graph neural networks (GNNs) with DRL, showing great potential, how to systematically address the unique multi-hop link congestion problem in this paradigm remains a challenge that requires further investigation. To systematically address these challenges, this paper is motivated by a series of interconnected research questions. We investigate how to effectively model the dynamic and topologically complex VEC network to capture global state information, particularly regarding inter-node dependencies and congestion, thus forming a solid foundation for intelligent decision-making. Building on this, we explore how to design a sophisticated decision-making mechanism capable of navigating the hybrid action space, which involves not only selecting a discrete offloading destination but determining continuous ratios for task offloading and resource allocation. Furthermore, we examine how to structure a learning framework guided by a multi-objective reward function to effectively balance the conflicting goals of minimizing task latency and mitigating network congestion. Ultimately, this study seeks to validate that such an integrated approach can yield a solution that is significantly more efficient, adaptive, and robust than traditional heuristic and standard reinforcement learning methods, especially under varying network scales and loads. A detailed review and analysis of these related works will be presented in Section 2. This paper proposes an intelligent, distributed task offloading algorithm based on graph attention policy optimization (GAPO). This algorithm deeply integrates the powerful representation learning capabilities of graph neural networks with the sequential decision-making abilities of deep reinforcement learning.

The main contributions of this paper are summarized as follows:

1. We propose a novel GNN-based state encoder that effectively captures global contextual information and spatiotemporal dependencies from the complex network topology, overcoming the problem of incomplete state information in traditional RL methods.
2. We design a novel attention-based policy network within an Actor–Critic framework that intelligently selects the optimal offloading destination while collaboratively determining the continuous ratios for offloading and resource allocation.
3. We develop a comprehensive multi-objective reward function that guides the agent to simultaneously minimize task completion latency and, crucially, to alleviate network congestion on multi-hop communication links.
4. Through comprehensive simulations and ablation studies, we demonstrate that GAPO significantly outperforms traditional heuristics and standard DRL approaches in both latency and congestion mitigation.

## 2. Related Work

The problem of task offloading in VEC has been approached from various angles, ranging from traditional optimization techniques to modern machine learning methods. This section provides a structured overview of the existing literature, contextualizing our proposed GAPO algorithm.

### 2.1. Traditional and Heuristic Approaches

Early attempts to solve the VEC offloading problem often relied on formal mathematical optimization. Many works have formulated the problem as a mixed-integer nonlinear programming (MINLP) problem, as seen in the work by Dai et al. [5] and Sahni et al. [6]. These formulations are comprehensive, capturing many system variables, but are generally NP-hard, making them computationally intractable for real-time decision-making in large-scale networks.

To find more tractable solutions, researchers have explored various optimization techniques. For instance, Han et al. employed semidefinite relaxation (SDR) to find an approximate solution for a heterogeneous offloading model where communication costs are asymmetric, providing a theoretical performance guarantee under certain conditions [7]. Other approaches have utilized Lyapunov optimization to design online algorithms that can make decisions without requiring knowledge of future system states, decomposing long-term objectives into a series of per-timeslot decisions [8]. While powerful for ensuring system stability, these methods may not fully capture the complex spatial dependencies inherent in multi-hop network topologies. Heuristic algorithms, such as the greedy lowest load method (one of our baselines), offer low-complexity solutions but often lead to suboptimal, myopic decisions that can cause network bottlenecks, such as the “hotspot” effect, where traffic is funneled towards a single powerful server. Other foundational approaches in intelligent vehicle systems have relied on different paradigms. For instance, some research has combined machine learning with heuristics, such as using a deep neural network to first classify vehicle states, with a subsequent heuristic algorithm making management decisions based on this learned information [9]. Another classic approach involves knowledge-based expert systems; for example, Zielonka et al. [10] developed a real-time diagnostic system using a type-2 fuzzy control system which relies on predefined expert rules to interpret sensor data. While effective for their specific, well-defined problems, these methods highlight the limitations that motivated our work. A system based on pre-set heuristics or fuzzy rules can be difficult to scale and adapt to the highly stochastic dynamics and complex topological nature of multi-hop VEC environments, a challenge our end-to-end DRL-based approach is explicitly designed to overcome.

These traditional and heuristic methods, while providing important foundational insights, often struggle with the high dimensionality, stochastic dynamics, and complex topological nature of multi-hop VEC environments.

### 2.2. Deep Reinforcement Learning for Task Offloading

To overcome the limitations of static models, deep reinforcement learning (DRL) has emerged as a powerful paradigm. DRL agents can learn effective policies through direct interaction with a dynamic environment, making them well-suited for VEC scenarios. Many DRL-based solutions have explored deep deterministic policy gradient (DDPG) or deep Q-networks (DQN) for resource allocation and for offloading decisions [11,12].

However, a significant limitation of these standard DRL approaches is their reliance on a “flattened state vector” to represent the network environment. This approach struggles to capture the complex topological structure and spatiotemporal dependencies among nodes. As we demonstrated in our ablation study (Section 5.3), this loss of topological information prevents the agent from perceiving multi-hop link congestion, leading to poor performance.

### 2.3. Emerging GNN-Based DRL Methods

Recognizing the limitations of flattened state representations, a recent and promising research trend is the integration of graph neural networks (GNNs) with DRL. GNNs are inherently designed to operate on graph-structured data, making them a natural fit for modeling complex network topologies and learning context-aware node representations [13,14].

Several studies have begun to explore this synergy. For example, Wu et al. proposed a heterogeneous graph attention network (HGAT) combined with DRL to handle dependency-aware task offloading, where tasks are modeled as directed acyclic graphs (DAGs) [15]. Their work excels at managing complex subtask dependencies. By contrast, our proposed GAPO algorithm focuses on a different but equally critical challenge: managing inter-node resource competition and mitigating backbone link congestion in a multi-hop environment. While both approaches leverage GNNs, their problem formulations and architectural focus differ, addressing complementary aspects of the VEC offloading problem. The core idea of GAPO is to use the GNN to learn a state representation that explicitly encodes network-wide congestion, enabling the DRL agent to make globally-aware, non-greedy decisions.

To further clarify our contribution, it is crucial to differentiate our work from GNN applications in related domains, such as the traffic forecasting model proposed by Polowczyk et al. [14] While both our work and theirs leverage GNNs to capture spatial dependencies in a network—be it communication links or city streets—the fundamental problem and technical approach are distinct. Polowczyk et al. focused on a predictive task: forecasting future traffic conditions. They ingeniously combined a GNN with an LSTM within a supervised learning framework to predict future values. Their model answers the following question: “What will the network state be?”

In stark contrast, GAPO addresses a sequential decision-making and control problem: task offloading in Vehicular Edge Computing. Our framework does not predict the future state; it learns a policy to take optimal actions in the current state. In our approach, the GNN functions as a powerful state encoder for a deep reinforcement learning (DRL) agent. Its role is to create a rich, context-aware representation of the current network environment, including computational loads and link congestion. This state representation is then fed into our attention-based Actor–Critic network, which answers the following question: “Given the current state, what is the best action to take now?” Our agent learns this decision-making process through trial-and-error interaction with the environment, guided by a reward signal, which is a fundamentally different paradigm from supervised forecasting.

Therefore, while Polowczyk et al. provided a valuable tool for traffic prediction, our work introduces a complete DRL-based control framework that utilizes GNN-encoded state information to actively manage network resources and to make complex, multi-objective offloading decisions in real-time.

### 2.4. Comparison with State-of-the-Art and GAPO’s Contributions

To further contextualize our work, we compare GAPO methodologically with two recent state-of-the-art solutions. F. Busacca et al. proposed MANTRA [16], a distributed framework using multi-armed bandits (MABs) where battery-powered RSUs (M-Boxes) autonomously manage their power states and offloading decisions. MANTRA’s MAB-based approach is highly effective for the discrete action problem of selecting an offloading target. However, GAPO addresses a more complex hybrid action space; our attention-based Actor network not only selects where to offload (a discrete action) but jointly determines how much to offload (a continuous ratio) and how much resource to allocate (another continuous ratio), providing a more fine-grained, integrated decision-making process.

S. Chen et al. [17] formulated the offloading problem as a multi-objective optimization problem (MOOP) and solved it using a novel hybrid genetic algorithm. Evolutionary algorithms like this are powerful for finding a set of Pareto-optimal solutions for a given state. By contrast, GAPO is designed for sequential, real-time decision-making. Our DRL framework learns a policy that maps the current network state to an immediate, effective action, which is inherently more adaptive to the step-by-step evolution of a dynamic VEC environment.

## 3. System Model

This section provides a detailed exposition of the system model for a multi-hop VEC network. It covers the network architecture, graph representation, task characteristics, latency models, and the offloading decision framework. Additionally, it presents the communication and computation models used to evaluate task processing latency and resource utilization. This section provides a detailed exposition of the system model for a multi-hop VEC network. It covers the network architecture, graph representation, task characteristics, latency models, and the offloading decision framework. To enhance clarity and to provide a convenient reference, the main notations used throughout this section are summarized in Table 1.

### 3.1. System Architecture

**Figure 1:**
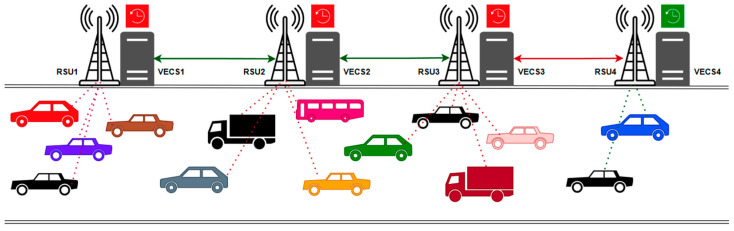
Multi-hop Vehicular Edge Computing Network.

We consider a multi-hop VEC network environment designed to provide low-latency, high-reliability computation services for mobile vehicles. As shown in Figure 1, the system architecture primarily consists of three core components: vehicles, roadside units (RSUs), and the communication links between them. As depicted in Figure 1, the scenario involves heterogeneous RSU loads, where RSU1–RSU3 serve more vehicles than RSU4. This imbalance can cause vehicles to offload tasks to the less-loaded RSU4, which in turn causes the transmission links related to RSU4 to become congested. GAPO aims to achieve lower link congestion rates while ensuring low latency.

We consider a one-way highway of length $L_{road}$. The vehicle layer is composed of a set of dynamically moving vehicles, denoted as $V=\left\{v_{1},v_{2},\ldots ,v_{N}\right\}$, where each vehicle $v_{n}\in V$ is treated as a mobile computation node. Vehicles are the primary initiators of computational tasks. These tasks are typically computation-intensive and latency-sensitive, such as path planning in real-time navigation, image rendering in augmented reality applications, or environmental perception data analysis in autonomous driving. Each vehicle $v_{n}$ is equipped with a limited on-Board unit (OBU) with a computational capacity denoted by $F_{v_{n}}^{local}$, measured in giga-cycles per second. Due to constraints in size, power consumption, and cost, the local computational capability of a vehicle is far inferior to that of dedicated edge servers. Vehicles are equipped with wireless communication modules, supporting communication with other vehicles (vehicle-to-vehicle, V2V) and with roadside infrastructure (vehicle-to-infrastructure, V2I), especially RSUs. The edge computing layer consists of a set of roadside units deployed along the road, denoted as $R=\left\{r_{1},r_{2},\ldots ,r_{M}\right\}$, where M is the total number of RSUs. RSUs act as bridges connecting vehicles to the core network and provide edge computing resources. Compared to the vehicles, each RSU $r_{m}\in R$ possesses significantly more powerful computing servers, with its computational capacity denoted as $F_{r_{m}}^{RSU}$. These RSUs can efficiently process computation-intensive tasks offloaded from the vehicles.

We consider the heterogeneity of the RSU computational capacity, meaning different RSUs may have different processing speeds, configured from a predefined set based on their deployment location and expected load. In this paper, we assume RSUs provide network access to vehicles within their coverage area via the IEEE 802.11p [18] wireless interface. RSUs are interconnected through a high-speed, low-latency backbone network, forming a multi-hop collaborative network. This RSU-to-RSU (R2R) connectivity allows for the forwarding and migration of tasks or their results between different RSUs, thereby enabling broader resource sharing and more flexible load balancing. However, for such multi-hop networks, the problem of link congestion becomes highly prominent. In our model, we assume the RSUs form a linear topology based on their geographical locations, and the R2R links have a base bandwidth of $B^{r2r}$.

### 3.2. Network Graph Representation

To effectively model and analyze the complex multi-hop VEC system, we abstract it into a dynamic, attributed graph structure. At any given time t, the network can be formally represented as a weighted graph $G_{t}=\left(N_{t},E_{t},X_{t},E_{t}\right)$. $N_{t}$ represents the set of nodes, which includes the set of all vehicles within the system’s service area at time t, $V_{t}=\left\{v_{1},v_{2},\ldots ,v_{N_{t}}\right\}$, and the set of RSUs, R. Here, $N_{t}$ is the number of active vehicles at that time. Due to vehicle mobility, $V_{t}$ is a time-varying set. $E_{t}$ represents the set of edges, which indicates the communication links existing between nodes at time t. We denote the V2R links between vehicles and RSUs as $E_{t}^{v2r}=\left\{\left(v_{n},r_{m}\right)|v_{n}\in V_{t},r_{m}\in R\right\}$. The establishment and termination of these links are directly affected by vehicle locations and RSU coverage ranges, and are therefore time-varying. The R2R links between RSUs are denoted as $E^{r2r}=\left\{\left(r_{i}{,r}_{j}\right)|r_{i}{,r}_{j}\in R\right\}$. These links are generally static, determined by the physical connections of the infrastructure. $X_{t}\in R^{\left|N_{t}\right|\times D_{N}}$ represents the node attribute matrix, where $D_{N}$ is the dimension of node features. For any node $u\in N_{t}$ its attribute vector $x_{u,t}\in R^{D_{N}}$ contains the following information:

1. Node type: A one-hot encoding vector indicating whether the node is a vehicle or an RSU.
2. Node computational capacity.
3. Current computational load: The instantaneous CPU utilization of the node, $\rho_{cpu}\left(u,t\right)$.
4. Available computational resources: The currently unallocated computational resources of the node.
5. Geographical location information: The coordinates of the node, which are dynamic for vehicles and fixed for RSUs.
6. Node degree: The number of connections the node has in the graph.

These attributes provide rich input for the subsequent GNN, enabling it to learn an effective representation of the node’s state.

$E_{t}\in \;R^{\left|E_{t}\right|\times D_{E}}$ represents the edge attribute tensor, where $D_{E}$ is the dimension of edge features. For any edge $e=\left(u,w\right)\in \;E_{t}$, its attribute vector $e_{u,w}\in \;R^{D_{E}}$ includes the following:

1. Link type: A one-hot encoding indicating whether it is a V2R or R2R link.
2. Link bandwidth capacity.
3. Current link load: The instantaneous utilization of the link, $\rho_{link}\left(e,t\right)$.
4. Communication latency: The estimated transmission latency of the link.
5. Link reliability: The packet loss rate of the link.

The key characteristic of this graph representation is its dual dynamicity. Vehicle mobility causes the V2R link topology $E_{t}^{v2r}$ and the vehicle node set $V_{t}$ to change continuously. Concurrently, the random arrival and completion of tasks lead to real-time fluctuations in the load attributes of nodes and links. This dual dynamicity poses significant challenges for task offloading decisions. First, the complexity of the state space, with a constantly changing network state, makes traditional optimization methods difficult to apply. Second, the demand for real-time decision-making requires rapid responses to network changes to ensure service quality. Finally, local decisions need to consider the state of the global network.

### 3.3. Task Processing and Communication Latency Model

In the system, each vehicle $v_{n}\in V$ dynamically generates a series of computational tasks. Each task $T_{i}≜\left(d_{i},c_{i},a_{i}\right)$ is treated as an independent computational unit, where $d_{i}$ is the data size of the task, $c_{i}$ is its computational complexity (measured in the number of CPU cycles required to complete the task), and $a_{i}$ is its arrival time. In this paper, task arrivals are modeled as a Poisson process.

For each task $T_{i}$, the generating vehicle $v_{n}$ must make the following decisions:

1. Offloading Destination Selection:

(1) $$K_{i}=\left\{\begin{array}{l} v_{n},\;if\;the\;task\;is\;processed\;locally\;on\;the\;vehicle\\ r_{m},\;if\;the\;task\;is\;offloaded\;to\;r_{m}\in \;R \end{array}\right.$$

2. Offloading Ratio:

If a task is offloaded, the agent can decide the ratio $\lambda_{i}\in \;\left[0,1\right]$ of the task’s computational workload to be offloaded.

The offloaded computation workload is as follows:

(2) $$C_{i}^{offload}=\lambda_{i}\cdot c_{i}$$

The locally processed workload is as follows:

(3) $$C_{i}^{local}=\left(1-\lambda_{i}\right)\cdot c_{i}$$

The amount of data transmitted to the RSU is as follows:

(4) $$D_{i}^{tx}=\lambda_{i}\cdot d_{i}$$

If $\lambda_{i}=0$, it represents local execution; if $\lambda_{i}=1$, it represents full offloading.

3. Computational Resource Allocation Ratio:

If a portion of the task ($\lambda_{i}>0$) is offloaded to RSU $r_{m}$, the agent also determines the proportion $\alpha_{i}\;\in \;\left[0,1\right]$ of $r_{m}$’s total computational capacity $F_{r_{m}}^{RSU}$ that will be dedicated to processing $C_{i}^{offload}$. This can be viewed as a form of resource reservation or priority scheduling based on slicing.

The allocated computational resource is as follows:

(5) $$F_{r_{m},i}^{alloc}=\alpha_{i}\cdot F_{r_{m}}^{RSU}$$

If the task is processed locally ($K_{i}=\;v_{n}$), we let $\lambda_{i}=0$ and $\alpha_{i}=1$ for notational convenience.

Therefore, the action $A_{i}$ that the agent selects for each task $T_{i}$ is represented by a tuple $A_{i}≜\left(K_{i},\lambda_{i},\alpha_{i}\right)$, where $K_{i}$ is the discrete offloading destination, and $\lambda_{i}$ and $\alpha_{i}$ are continuous ratios for offloading and resource allocation, respectively.

Communication latency depends on the link type and its current load. In this study, we model the dynamic queuing behavior of V2R and R2R links. While the system conceptually aligns with a G/G/1 queue due to its general arrival and service patterns, we adopt specific, standard models for simulation and latency calculation to ensure reproducibility. Specifically, the arrival process is modeled as a Poisson process. This is implemented in our simulation by generating the inter-arrival times between consecutive tasks from an exponential distribution. For the service time and latency estimation, we use the well-known and computationally efficient M/M/1 queuing formula as a practical approximation. In our simulation, a task’s data size and computational requirement are drawn from uniform distributions. The base service time is then calculated deterministically from these values. We use this service time within the M/M/1 latency formula to estimate the total time in the system, which includes queuing delay. This approach effectively captures the essential non-linear relationship where latency increases sharply with utilization, a critical feature for modeling network congestion, while remaining tractable for a dynamic simulation environment. The use of the M/M/1 model is a deliberate methodological choice, justified on the following grounds: The M/M/1 model provides a tractable, closed-form expression for calculating latency. This is computationally essential within our deep reinforcement learning framework, where the agent must rapidly evaluate the consequences of numerous potential actions during the training process. A full-scale discrete-event simulation of a G/G/1 queue at each step would render the training of our complex agent computationally infeasible. While an approximation, the M/M/1 model effectively captures the core non-linear relationship between resource utilization and delay. It correctly reflects the critical phenomenon that latency increases exponentially as a link or the server approaches its full capacity. This is the primary system dynamic that our learning agent must understand and manage to avoid congestion. While an approximation, the M/M/1 model effectively captures the core non-linear relationship between resource utilization and delay. It correctly reflects the critical phenomenon that latency increases exponentially as a link or server approaches its full capacity. This is the primary system dynamic that our learning agent must understand and manage to avoid congestion. For V2R links, we treat each as a G/G/1 queue. Each RSU $r_{m}\in R$ has a total V2R wireless interface bandwidth, denoted as $B_{r_{m}}^{v2r}$. This bandwidth is limited and must be dynamically shared among all vehicles currently connected to and transmitting data to that RSU. From a macroscopic perspective, this means the effective data transmission rate for a single vehicle decreases as the number of competing vehicles increases.

For a specific vehicle $v_{n}$ transmitting data to RSU $r_{m}$, its instantaneous data rate $R_{v_{n},r_{m}}^{v2r}$ depends not only on the RSU’s total bandwidth $B_{r_{m}}^{v2r}$ but on the channel conditions and the number of vehicles currently sharing the bandwidth. We consider a total link service capacity and treat all data flows arriving at this link as entering a shared queue. When vehicle $v_{n}$ needs to transmit data of size $D_{i}$ to RSU $r_{m}$, the transmission latency is mainly composed of transmission time and queuing time.

1. Transmission Time:

(6) $$L_{tx}=\frac{D_{i}}{R_{eff}}$$

where $R_{eff}$ is the effective data rate obtained for this transmission.

2. Queuing Time:

In this study, to obtain a computable latency estimate, we use the classic M/M/1 queuing formula as an approximation for the G/G/1 model’s performance [19]. This is a common practice in engineering that preserves the core idea of queuing theory (i.e., latency increases non-linearly with utilization) while providing an easy-to-compute closed-form solution. The V2R interface of RSU $r_{m}$ is viewed as a single server with a total service capacity of $B_{r_{m}}^{v2r}$ (bps). All vehicles wishing to upload data via this RSU form an arrival flow. Let $Q_{arrival}^{v2r,m}$ be the current total data arrival rate for the V2R uplink at RSU $r_{m}$, which is estimated using an exponentially weighted moving average (EWMA) of recent task arrival contributions. If $Q_{arrival}^{v2r,m}$ is greater than or equal to the effective service rate, the link is considered highly congested, and latency will increase sharply. The total V2R bandwidth $B_{r_{m}}^{v2r}$ of the RSU is considered the service rate of this queue.

Therefore, the V2R communication latency for transmitting data of size $D_{i}$, $L_{v2r}\left(D_{i},r_{m}\right)$, is calculated as follows:

(7) $$L_{v2r}\left(D_{i},r_{m}\right)=\frac{T_{s}^{v2r}}{1-\rho_{link}^{v2r,m}+\epsilon }$$

where $T_{s}^{v2r}=\;\frac{D_{i}}{B_{r_{m}}^{v2r}+\epsilon }$ is the basic service time without considering queuing, $\rho_{link}^{v2r,m}$ is the actual utilization of this V2R link, a factor less than 1 used to ensure queue stability and to reflect that real systems cannot achieve 100% utilization, and ϵ is a very small value to prevent division by zero.

To provide the agent with more fine-grained information that reflects instantaneous channel competition, we use a different calculation for the RSU’s V2R link utilization $\rho_{link}^{v2r,m}$ when constructing the node features for the GNN. Specifically, when generating the node attribute vector $X_{t}$, this utilization is calculated as the ratio of the total arrival rate to an effective service rate adjusted by the number of competing vehicles. This allows the GNN to perceive the drop in effective bandwidth due to an increase in connected vehicles, thereby making more forward-looking offloading decisions.

R2R links typically use more stable and higher-bandwidth technologies than V2R links, such as fiber optic connections or dedicated point-to-point high-frequency wireless links. This means R2R links usually have higher data transmission rates and lower intrinsic latency. For a specific R2R link connecting RSU $r_{a}$ and $r_{b}$, its base bandwidth capacity is denoted as $B_{r_{a},r_{b}}^{r2r}$. Unlike V2R links, the traffic on R2R links is often bidirectional and may carry data for multiple different tasks simultaneously. When data of size $D_{i}$ needs to be transmitted between RSU $r_{a}$ and $r_{b}$ via a direct R2R link, its communication latency $L_{r2r}\left(D_{i},r_{a},r_{b}\right)$ is modeled similarly to V2R, also as a G/G/1 queue, with latency calculated approximately as follows:

(8) $$L_{r2r}\left(D_{i},r_{a},r_{b}\right)=\frac{T_{s}^{r2r}}{1-\rho_{link}^{r_{a},r_{b}}+\epsilon }$$

where $T_{s}^{v2r}=\;\frac{D_{i}}{B_{r_{a},r_{b}}^{r2r}+\epsilon }$ is the basic service time, and $\rho_{link}^{r_{a},r_{b}}$ is the utilization of this R2R link.

If data needs to be transmitted through a series of RSUs via multi-hop, for example, along the path $P_{R2R}\;=\;\left(r_{s},\;r_{1},\;r_{2}\ldots ,\;r_{k},r_{d}\right)$, where $r_{s}$ is the source RSU and $r_{d}$ is the destination RSU, the total multi-hop R2R communication latency is the sum of the latencies of each link segment along the path, as follows:

(9) $$L_{multi-hop}\left(D_{i},P_{R2R}\right)=\sum_{\left(r_{u},r_{v}\right)\in P_{R2R}}L_{r2r}\left(D_{i},r_{u},r_{v}\right)$$

To accurately reflect the dynamic load conditions of the network links, the data arrival rates on V2R and R2R links need to be continuously updated. The system employs an exponentially weighted moving average (EWMA) mechanism. When a data block of size d is assigned to a link, it contributes to that link’s average arrival rate over a time window. As time passes, the contribution of old arrivals decays exponentially. After each time step Δt, the current arrival rate is multiplied by a decay factor. This dynamic update mechanism allows the model to capture changes in network congestion over time, providing more accurate link status information for the agent’s decisions.

In our G/G/1 queuing model, when the arrival rate β of a communication link approaches or exceeds its effective service rate μ, the queuing latency of that link increases sharply, and the system becomes unstable. We define this state as link congestion. Specifically, we introduce a stability factor φ < 1. When the link utilization ρ=β/μ satisfies ρ≥φ, we determine that the link is congested. Specifically, we introduce a stability factor φ < 1, set to 0.99 in our simulations (as shown in Table 2). This factor represents a crucial engineering principle: real-world queuing systems become unstable and experience exponential latency growth before reaching 100% theoretical utilization. By defining congestion as occurring when utilization ρ exceeds this factor, we provide a practical and safe margin to prevent system collapse and to guide the agent to learn proactively to avoid such states. The severity of congestion can be measured by the proportion by which its actual utilization exceeds this stability threshold. Alleviating link congestion is a core optimization objective of this research. To determine the end-to-end communication latency from a vehicle to any potential target RSU, the optimal path that minimizes total latency must be found. This involves selecting an initial RSU to act as a wireless access point and then finding the lowest-latency multi-hop path through the RSU backbone network to the final destination. The procedure for computing this optimal path latency is formalized in Algorithm 1.



**Algorithm 1: Multi-Hop Path Latency Calculation**

**Input:**

$\;\mathrm{source}\;\mathrm{vehicle}\;v_{n}$


$,\;\mathrm{target}\;\mathrm{RSU}\;r_{m}$


$,\;\mathrm{task}\;\mathrm{data}\;\mathrm{size}\;D_{i}$


**Output:**

$\;\mathrm{best}\;\mathrm{path}\;P^{*}$


$,\;\mathrm{minimum}\;\mathrm{latency}\;L^{*}$

$1:\;\mathrm{Initialize}\;L^{*}\;\leftarrow \;\infty$, $P^{*}$ ← None


$2:\;R_{access}\;\leftarrow \;\mathrm{Get}\;\mathrm{all}\;\mathrm{RSUs}\;\mathrm{directly}\;\mathrm{connected}\;\mathrm{to}\;\mathrm{vehicle}\;v_{n}$



$3:\;\mathrm{Create}\;\mathrm{RSU}-\mathrm{only}\;\mathrm{subgraph}\;G_{r}$

$4:\;\mathrm{for}\;\mathrm{each}\;r_{access}$$\;\mathrm{in}\;R_{access}$ **do**$5:\;L_{v2r}\;\leftarrow \;\mathrm{Get}\;V2R\;\mathrm{Latency}\;(v_{n}$$,\;r_{access}$$,\;D_{i}$)
$6:\;\mathrm{if}\;r_{access}$$\;\mathrm{is}\;r_{m}$ **then**

$7:\;L_{path}$


$\;\leftarrow \;L_{v2r}$

$8:\;P_{c}$$\leftarrow \;[v_{n}$$,\;r_{m}$]
9:            **else**
$10:\;\mathrm{Define}\;\mathrm{edge}\;\mathrm{weights}\;\mathrm{in}\;G_{r}$ as R2R latency:
$11:\;\mathrm{for}\;\mathrm{each}\;\mathrm{edge}\;(r_{u},r_{v}$$)\;\mathrm{in}\;G_{r}$ **do**

12:      W


$(r_{u},r_{v}$


$)\;\leftarrow \;\mathrm{Get}\;R2R\;\mathrm{Latency}\;(D_{i},r_{u},r_{v}$


$)\;\mathrm{for}\;\mathrm{all}\;\mathrm{edges}\;\mathrm{in}\;G_{r}$

13:      **end for**
$14:\;P_{v2r}$$\;\leftarrow \;\mathrm{Shortest}\;\mathrm{Path}\;(G_{r}$$,\;\mathrm{source}\;=\;r_{access}$$,\;\mathrm{target}\;=\;r_{m}$, weights = W)
$15:\;\mathrm{if}\;P_{v2r}$ exists **then**


$16:\;L_{r2r}$


$\;\leftarrow \;\mathrm{Calculate}\;\mathrm{total}\;\mathrm{latency}\;\mathrm{of}\;P_{v2r}$


 using weights W



$17:\;L_{path}$


$\;\leftarrow \;L_{v2r}$


$\;+\;L_{r2r}$

$18:\;P_{c}$$\leftarrow \;[v_{n}$] ⊕$P_{r2r}$ // ⊕ is path concatenation
19:      **else**
20:        **continue**
21:      **end if**
22:          **end if**
$23:\;\mathrm{if}\;L_{path}$$<\;L^{*}$ **then**

$24:\;L^{*}$


$\;\leftarrow \;L_{path}$



$25:\;P^{*}$


$\;\leftarrow \;P_{c}$

26:          **end if**
27:  **end for**


$28:\mathrm{return}\;P^{*}$


$,\;L^{*}$




### 3.4. Computation Model and Total Latency Analysis

When vehicle $v_{n}\in V$ decides to process task $C_{i}^{local}$ locally, its computation latency primarily depends on the vehicle’s own computational capacity and its current computation queue state. Each vehicle $v_{n}$ has a fixed local computational capacity, denoted as $F_{v_{n}}^{local}$. The vehicle’s local processing unit can be viewed as a server, and all task segments needing local processing form a computational task arrival flow. We similarly model this as a G/G/1 queue to approximately estimate the local computation latency. The local computation latency for a workload of $L_{local}\left(C_{i}^{local},v_{n}\right)$, is calculated as follows:

(10) $$L_{local}\left(C_{i}^{local},v_{n}\right)=\frac{T_{s}^{local}}{1-\rho_{cpu}^{v_{n}}+\epsilon }$$

where $T_{s}^{local}=\;\frac{C_{i}^{local}}{F_{v_{n}}^{local}+\epsilon }$ is the basic computation service time without queuing, $\rho_{cpu}^{v_{n}}$ is the actual utilization of vehicle $v_{n}$’s computation unit, and ϵ has the same meaning as in the communication model.

When task $C_{i}^{offload}$ is offloaded to RSU $r_{m}$ for processing, the computation latency depends on the RSU’s capacity, its overall current computational load, and the computational resources allocated to this specific task. In our model, the agent also outputs a computational resource allocation ratio $\alpha_{i}\;\in \;\left[0,1\right]$. Therefore, for the task portion using dedicated computational resources, its computation latency on RSU $r_{m}$, $L_{edge}\left(C_{i}^{offload},r_{m},\alpha_{i}\;\right)$, can be directly calculated as follows:

(11) $$L_{edge}\left(C_{i}^{offload},r_{m},\alpha_{i}\right)=\frac{C_{i}^{offload}}{F_{r_{m},i}^{alloc}+\epsilon }=\frac{C_{i}^{offload}}{\alpha_{i}\cdot F_{r_{m}}^{RSU}+\epsilon }$$

Although this part of the computation uses specifically allocated resources, it still consumes the RSU’s actual computational capacity. Therefore, when calculating the overall arrival rate of computation for RSU $r_{m}$, this offloaded workload $C_{i}^{offload}$ will still contribute to the RSU’s average computational load. This means that even if a task obtains a dedicated resource slice, it still affects the background load perceived by subsequent tasks on that RSU, which will be reflected in the node features observed by the GNN.

Total task latency is a key metric for evaluating system performance. For a given task $T_{i}\;≜\;\left(d_{i},\;c_{i},a_{i}\right)$, its total processing latency $L_{i}^{total}$ depends on the offloading decision $\left(K_{i},\lambda_{i},\;\alpha_{i}\right)$ made by the agent. The arrival time of task $T_{i}$ is $a_{i}$, and its completion time is $a_{i}+L_{i}^{total}$.

Based on the offloading decision model defined above, we analyze the total task latency in different scenarios:

1. Fully Local Execution:

When the task is processed entirely on the source vehicle $v_{n}$, the input data $d_{i}$ does not need to be transmitted, and the computation workload is $c_{i}$. The total latency is the local computation latency, which is calculated as follows:

(12) $$L_{i}^{total}=L_{local}\left(C_{i}^{local},v_{n}\right)$$

2. Full Offloading to an RSU:

When the task is fully offloaded to RSU $r_{m}$, the vehicle must first transmit the input data of size $d_{i}$ via V2R and potentially R2R multi-hop links to RSU $r_{m}$. Subsequently, RSU $r_{m}$ uses the allocated computational resources $F_{r_{m},i}^{alloc}=\;\alpha_{i}\;\cdot F_{r_{m}}^{RSU}$ to process the entire computation workload $c_{i}$. The total latency includes V2R communication latency, R2R multi-hop communication latency (if any), and RSU computation latency as follows:

(13) $$L_{i}^{total}=L_{v2r}\left(d_{i},r_{m}\right)+L_{multi-hop}\left(d_{i},P_{R2R}\right)+L_{edge}\left(c_{i},r_{m},\alpha_{i}\right)$$

3. Partial Offloading:

When the task is partially offloaded, its computational workload is split into a local part $C_{i}^{local}$ and an offloaded part $C_{i}^{offload}$. The data $D_{i}$ needs to be transmitted to the RSU. The local computation and the offloaded computation can be performed in parallel. Therefore, the total task latency is determined by the longer of the two paths as follows:

(14) $$L_{i}^{total}=max\left(L_{local}\left(C_{i}^{local},v_{n}\right),L_{v2r}\left(D_{i},r_{m}\right)+L_{multi-hop}\left(D_{i},P_{R2R}\right)\;+L_{edge}\left(C_{i}^{offload},r_{m},\alpha_{i}\right)\right)$$

Based on the preceding system architecture, task model, communication model, and computation model, the core problem of our research can be formulated as jointly optimizing the task offloading decisions and resource allocation strategies for a series of incoming tasks $\left\{T_{1},T_{2},\ldots ,T_{k}\right\}$ in a dynamic multi-hop VEC network, in order to minimize a comprehensive performance metric. This metric typically includes average task processing latency, load balancing among RSUs, and network congestion levels.

Assuming the system needs to process *K* tasks over a time horizon, for each task $T_{i}\left(i=1,2,\ldots ,K\right)$, the decision variables are $X={\left\{\left(K_{i},\lambda_{i},\alpha_{i}\right)\right\}}_{i=1}^{K}$. Our goal is to find the optimal set of decisions $X^{*}$ that minimizes a weighted multi-objective function.

The primary optimization objectives are as follows:

1. Minimize Average Task Processing Latency, $L_{avg}:$

(15) $$\underset{X}{\min}L_{avg}=\frac{1}{K}\sum_{i=1}^{K}L_{i}^{total}$$

2 Minimize Average R2R Link Congestion, $C_{cong}:$

This objective aims to penalize offloading decisions that cause excessive utilization of communication links. As defined in Section 3.3, congestion occurs when a link’s utilization $\rho_{link}$ exceeds the stability factor φ. We define the congestion level $C_{cong}$ as the sum of the average excess utilization over all links involved in task transmissions. For a set of paths $P_{\pi }$ generated by a policy π, this objective can be expressed as follows:

(16) $$\underset{X}{\min}C_{cong}=\frac{1}{\left|P_{\pi }\right|}\sum_{e\in P_{\pi }}\bar{\rho_{link}^{e}}-\varphi$$

where $\bar{\rho_{link}^{e}}$ is the average utilization of link e during the evaluation period. This objective encourages traffic dispersion, avoiding over-reliance on a few R2R links, and thus alleviating potential backbone network bottlenecks.

By introducing weight factors $w_{1}$, $w_{2}$ ≥ 0 (set to 1.0 and 0.5, respectively, in our experiments to prioritize latency while penalizing congestion), the above multiple objectives can be combined into a single scalar optimization objective function $J\left(Χ\right)$. These weights allow a network operator to balance the critical trade-off between performance and stability. A higher $w_{1}$ prioritizes minimizing task completion time, whereas a higher $w_{2}$ enforces a more conservative policy that prioritizes avoiding network congestion, even at the cost of slightly higher latency for some tasks.

(17) $$\underset{X}{\min}J\left(X\right)=w_{1}\cdot L_{avg}+w_{2}\cdot C_{cong}$$

$$s.t\;K_{i}\in \left[v_{i}\right]\cup R\forall i\in N$$

$$\lambda_{i}\in \left[0,1\right]\forall i\in N$$

$$\alpha_{i}\in \left[0,1\right]\forall i\in N$$

$$\rho_{cpu}^{r_{m}},\rho_{link}^{e}\in \left[0,100\right]$$

$$\beta_{e}\leq \varphi \cdot \mu_{e}\forall e\in E$$

$$X={\left\{\left(K_{i},\lambda_{i},\alpha_{i}\right)\right\}}_{i=1}^{K}$$

Formulating this problem as an optimization problem highlights its inherent complexity and challenges. The problem is NP-hard. Specifically, the formulation requires making discrete offloading decisions while optimizing the objectives and satisfying the constraints that are non-linear functions of the decision variables. This combination of discrete variables and non-linear, non-convex relationships places the problem in the class of non-convex MINLP, which is proven to be NP-hard [5]. The network state is dynamically changing over time, and may involve uncertainty, which makes traditional offline optimization methods inapplicable. Each decision affects future network states, exhibiting the characteristics of a Markov decision process (MDP). As the number of vehicles and RSUs increases, the state and action spaces grow dramatically.

## 4. Graph Attention Policy Optimization (GAPO)

This chapter provides a detailed introduction to our proposed GAPO algorithm, designed to solve the multi-objective task offloading and resource allocation problem in dynamic multi-hop VEC networks. We will first provide an overview of GAPO’s overall framework, then elaborate on its key components, including the graph representation of the network state and GNN embeddings, the architecture of the attention-based Actor–Critic policy network, and the design of the policy optimization module.

### 4.1. Overview of the GAPO Algorithm Framework

As described in Section 3, multi-hop VEC environments are characterized by high dynamics and complexity: the rapid movement of vehicles leads to frequent changes in wireless channel conditions and network topology; RSU resources are limited and may be heterogeneous; and task offloading decisions must consider not only the latency of individual tasks but the overall system’s load balancing and network congestion, with these objectives often being in conflict.

**Figure 2:**
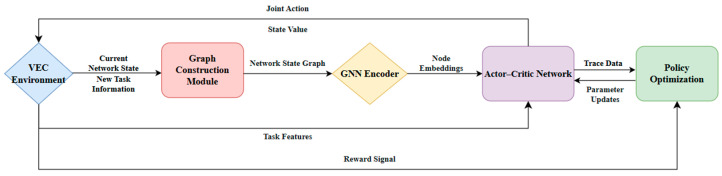
The GAPO Algorithm Framework.

Based on these considerations, this paper proposes an algorithm based on graph attention policy optimization (GAPO), which aims to solve the dynamic, multi-objective, and joint optimization problem of task offloading and resource allocation in multi-hop VEC networks. The core idea of the GAPO algorithm is to deeply integrate the powerful graph representation capabilities of graph neural networks (GNNs) with the intelligent decision-making abilities of deep reinforcement learning (DRL), and to guide the agent to learn an effective balance between multiple performance objectives through a well-designed reward mechanism. The overall framework of the GAPO algorithm is shown in Figure 2.

At each decision-making moment, when a new computational task is generated, the VEC environment provides the agent with the current network state and new task information. The network state includes the real-time attributes of all vehicles and RSUs, as well as their connection relationships and link quality. The new task information describes the characteristics of the task to be processed. The received raw network state and task information are fed into a Graph Construction Module. This module is responsible for integrating this scattered information into a structured network state graph. In this graph, vehicles and RSUs are abstracted as nodes, and the communication links between them are abstracted as edges. Each node and edge is endowed with a feature vector extracted from the raw state, which may have been normalized.

The constructed network state graph is then input into a GNN encoder. The GNN encoder processes the graph data through its internal graph convolutional layers. By propagating and aggregating neighbor information on the graph, the GNN can generate a low-dimensional, dense node embedding vector for each node in the graph. These embedding vectors not only capture the intrinsic attributes of the nodes but incorporate contextual information from their network topology, thereby providing a state representation with a more global perspective for subsequent decision-making. Concurrently, the GNN encoder also outputs a state value for the network state graph, which is a preliminary assessment of the quality of the current state and can be used for subsequent advantage calculation.

The node embeddings output by the GNN encoder, along with the current task features obtained directly from the VEC environment, are fed into the Actor–Critic network. The Actor–Critic network contains two core components: the Actor and the Critic. The Actor network generates a joint action based on the received state representation. This joint action includes the discrete selection of an offloading destination and the continuous decision of resource allocation. The generated joint action is then applied back to the VEC environment, which executes the task offloading and processing according to this action and transitions to the next state. After executing the action, the VEC environment returns a reward signal to the agent. This reward signal is calculated based on a predefined multi-objective reward function, comprehensively considering performance indicators, such as task processing latency, RSU load balancing, and network congestion.

Simultaneously, the entire interaction process generates a piece of experience data, known as a trajectory, which is stored in the experience replay buffer for policy optimization. The agent periodically samples a batch of experience data from the replay buffer. Using this data, it updates the parameters of the Actor–Critic networks. The Critic network learns to more accurately estimate the state value, while the Actor network learns to optimize its policy to maximize the cumulative expected reward.

Through the iterative steps described above, the GAPO algorithm can learn end-to-end. The GNN encoder learns how to extract effective feature representations from complex network states, while the Actor–Critic network learns how to make intelligent, multi-objective optimized decisions for task offloading and resource allocation in a highly dynamic and variable multi-hop VEC environment based on these representations.

### 4.2. GNN Network and Embeddings

As detailed in Section 3.2, we introduced the network graph representation. The attributed graph $G_{t}$ constructed in this manner dynamically reflects the real-time state of the VEC network and serves as the input to the GNN encoder.

**Figure 3:**
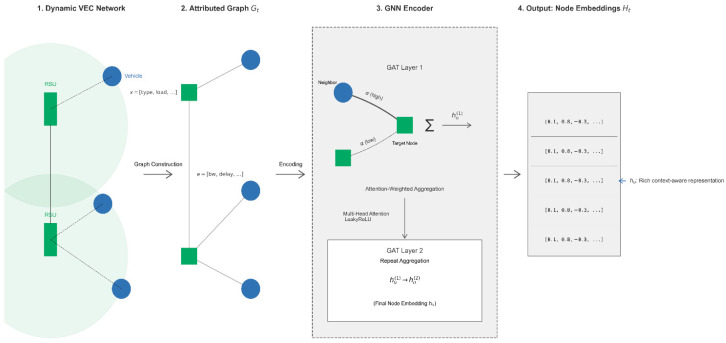
GNN Network and Embeddings.

As shown in Figure 3, the core task of the GNN encoder is to learn a mapping function $F_{GNN}:G_{t}\to H_{t}$, which transforms the input network state graph $G_{t}$ into a set of node embeddings, represented by the matrix $H_{t}\in R^{\left|N_{t}\right|\times D_{emb}}$ is the dimension of the node embeddings. The embedding vector for each node, $h_{u}\in R^{D_{emb}}$, should encode the properties of the node itself and its contextual information within the network.

In our GNN encoder, we employ a more powerful variant of the graph attention network, known as GATv2 [20], to enhance the model’s expressive power. Unlike the original GAT, which utilizes a static attention mechanism, GATv2 introduces a dynamic attention mechanism that allows the model to learn more complex and context-dependent relationships between nodes. This allows it to selectively aggregate the most important neighbor information for the current task. Even if the neighbor types and feature distributions of a node vary, GATv2 can adaptively learn how to aggregate their information. Moreover, GATv2’s operations are local, making it suitable for dynamically changing graph structures.

Our GNN encoder is composed of two stacked GATv2 convolutional layers. The first GATv2 layer employs multiple independent attention heads in parallel and concatenates their outputs to capture neighborhood relationships from different aspects, enhancing the model’s expressive power and stability. A LeakyReLU activation function is used to introduce non-linearity. Batch normalization is applied to the concatenated features after the first GATv2 layer and before the activation function to accelerate convergence and to improve generalization. It operates on the feature dimension. Additionally, dropout is applied to the attention weights and node features to prevent overfitting. Self-loops are used to ensure that a node’s own information is considered during aggregation. The second GATv2 layer typically sets the number of attention heads to 1 and directly outputs the final embedding dimension, as this layer is directly followed by the MLP layers of the Actor–Critic network, thus no activation function is designed for it.

In each GATv2 layer l, the representation of a node u is computed using a multi-head attention mechanism. For a single attention head k, the process is as follows:

First, an attention score $e_{uv}^{\left(k\right)}$ is calculated for each neighbor v in the neighborhood of u, as follows:

(18) $$e_{uv}^{\left(k\right)}={\left(a^{\left(k\right)}\right)}^{T}\cdot LeakyReLU\left(W^{\left(k\right)}\cdot \left[\left.h_{u}^{\left(l-1\right)}\right\|h_{v}^{\left(l-1\right)}\right]\right)$$

where $h_{u}^{\left(l-1\right)}$ is the feature vector of node u from the previous layer, $W^{\left(k\right)}$ and $a^{\left(k\right)}$ are the learnable weight matrix and attention vector for head k, respectively, and ∥ denotes concatenation. These scores are then normalized across all neighbors using the Softmax function to obtain the attention weights $a_{uv}^{\left(k\right)}$, as follows:

(19) $$a_{uv}^{\left(k\right)}=\frac{exp\left(e_{uv}^{\left(k\right)}\right)}{\sum_{j\in N\left(u\right)\cup \left\{u\right\}}exp\left(e_{uj}^{\left(k\right)}\right)}$$

Finally, the attention weights are used to aggregate the transformed features of the neighbors, producing the output for head k. For intermediate layers with k heads, we concatenate their outputs as follows:

(20) $$h_{u}^{\left(l\right)}={∥}_{k=1}^{k}\sigma \left(\sum_{v\in N\left(u\right)\cup \left\{u\right\}}a_{uv}^{\left(k\right)}\cdot \left(W^{\left(k\right)}h_{v}^{\left(l-1\right)}\right)\right)$$

where σ is a non-linear activation function. This dynamic attention mechanism is crucial for capturing the complex dependencies in the VEC network.

After passing through two GATv2 layers of propagation and aggregation, the final output node embedding $h_{u}=\;h_{u}^{\left(2\right)}$ captures the structural and feature information within a two-hop neighborhood centered on node u. These node embeddings are then fed into the Actor–Critic network as the basis for their subsequent decision-making and evaluation.

The core advantage of GATv2 lies not only in its powerful representation learning capability but in its inherent interpretability. In our model, each GATv2 layer performs an attention-weighted aggregation of neighbor information. This means that, when a node updates its own feature embedding, it does not treat all neighbor nodes and its own attributes equally. Specifically, each node has key features, such as current computational load and V2R link congestion. Through end-to-end training, GATv2 learns an attention function that automatically assigns higher attention weights to neighbor nodes with more favorable resources. For example, when an RSU aggregates information, it will pay more attention to its own low computational load features and the uncongested R2R links connected to it. After several layers of GATv2 information propagation, the final output node embedding is no longer an isolated feature vector but a highly condensed, context-aware representation of its own state and the availability of surrounding network resources. An RSU with low computational load and low communication congestion will have a final embedding vector that is significantly different from one in a “hotspot” area. This differentiated information aggregation, achieved through attention weights, is precisely the foundation for GAPO’s ability to understand the global network state and to make intelligent decisions, and it also provides a transparent window for us to understand the internal working mechanism of the model.

In this way, the GAPO algorithm uses GNNs to effectively compress raw, high-dimensional, and structured network state information into low-dimensional, dense, and context-rich node embeddings, providing a high-quality state representation for subsequent reinforcement learning decisions.

### 4.3. Attention-Based Actor–Critic Policy Network

In the GAPO algorithm, we employ the Actor–Critic reinforcement learning framework to learn the optimal task offloading and resource allocation policy. This framework consists of two core neural networks: the Actor network and the Critic network. These two networks share the node state embeddings extracted by the underlying GNN encoder and perform their respective computations based on this foundation. This section will detail the structure and function of the Actor and Critic networks.

**Figure 4:**
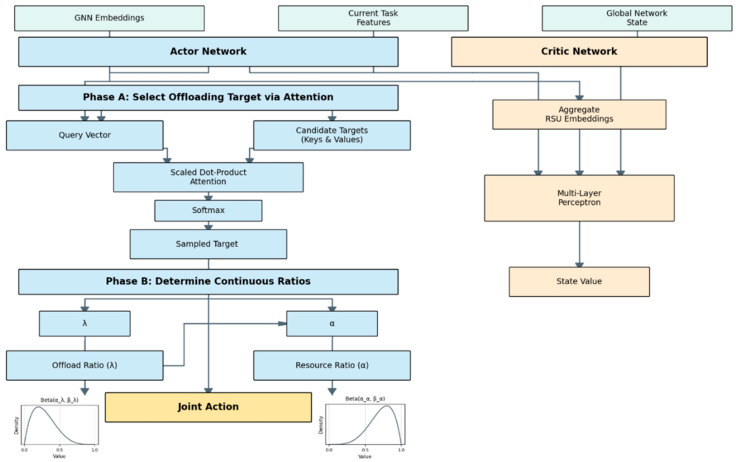
Attention-based Actor–Critic Policy Network.

As shown in Figure 4, the Actor network is responsible for formulating a specific action policy based on the current environmental state and task information. For each task to be processed, the Actor network needs to output a joint action, which includes the discrete selection of an offloading destination and two continuous resource allocation ratios (offloading ratio and computational resource allocation ratio). The inputs to the Actor network are the GNN node embeddings $h_{u}$ and the current task feature vector $X_{task}$. For the discrete action of selecting an offloading destination, the Actor network employs a scoring and selection module based on an attention mechanism. First, a candidate target embedding matrix $C_{target}\;\in \;R^{\left(1+M_{slot}\right)\times D_{emb}}$ is constructed, where $M_{slot}$ is the number of fixed slots defined for RSUs in the observation space. The first row corresponds to the embedding of the source vehicle, $h_{veh}$. The subsequent $M_{slot}$ rows correspond to the embeddings of the various RSU slots. If an RSU slot is invalid in the current observation, its corresponding embedding can be set to a zero vector or to a special marker vector, and it will be masked out in the subsequent attention calculation. To intelligently select the offloading destination, the Actor network uses a query-based attention mechanism, which can be intuitively understood as an “intelligent matching” process. First, the model concatenates the GNN embedding of the current task’s source vehicle $h_{veh}$, the task features $X_{task}$, and the global network state $g_{stats}$, and then transforms this into a query vector through a small MLP network as follows:

(21) $$q_{att}={MLP}_{query}\left(\left[h_{veh}⊕X_{task}⊕g_{stats}\right]\right)$$

where ${MLP}_{query}$ is a small multi-layer perceptron, with an output dimension typically identical to the node embedding dimension $D_{emb}$, and uses a Tanh activation function, and ⊕ denotes vector concatenation. This query vector can be understood as a concise representation of the current decision-making needs, i.e., for this specific task, considering the current overall network environment, what characteristics should an ideal offloading destination have. The GNN embeddings of the candidate targets, $c_{j}\in \;C_{target}$, form the set of targets. As mentioned earlier, the GNN encoder has already encoded the resource status and topological context of each node into these embedding vectors. Therefore, the embedding $c_{j}$ of an RSU with low load and high computational power naturally becomes a high-quality target for the aforementioned “query”. The Actor network calculates an attention score $e_{j}$ by computing the scaled dot-product similarity between the query vector $q_{att}$ and each candidate answer as follows:

(22) $$e_{j}=\frac{q_{att}\cdot c_{j}^{T}}{\sqrt{D_{emb}}}$$

An RSU with ideal characteristics will have its embedding vector $c_{j}$ be more “aligned” or “similar” to the query vector $q_{att}$ in the high-dimensional space, thus receiving a higher attention score. For invalid candidate targets, their attention scores $e_{j}$ are set to negative infinity, so that their probability will approach zero in the subsequent Softmax operation.

The calculated attention scores are converted into a probability distribution for selecting each offloading destination, $P_{target}$, using a Softmax function as follows:

(23) $$P_{target}=Softmax\left(e\right)$$

where $e=\left[e_{0},e_{1},\ldots ,e_{M_{slot}}\right]$. This is a Categorical distribution, and the agent will sample a discrete action $K_{i}\in \left\{0,1,\ldots ,M_{slot}\right\}$ from this distribution, representing the selected offloading destination (0 for local, 1 to $M_{slot}$ for the corresponding RSU slot).

After determining the offloading destination $K_{i}$, the Actor network needs to further decide on two continuous ratio values. For the offloading ratio, the input is the concatenated vector $\left[h_{veh}⊕{h_{target}⊕X}_{task}⊕g_{stats}\right]$. An independent MLP network maps this input to the two parameters $\left(a_{\lambda },b_{\lambda }\right)$ of a Beta distribution. These two parameters are typically passed through a Softplus activation function to ensure they are positive and greater than or equal to 1, to maintain the good shape of the Beta distribution. The offloading ratio $\lambda_{i}$ is then sampled from the $Beat\left(a_{\lambda },b_{\lambda }\right)$ distribution. For the computational resource allocation ratio, the input is the concatenated vector $\left[h_{veh}⊕{h_{target}⊕X}_{task}⊕g_{stats}⊕\lambda_{i}\right]$. Here, the sampled offloading ratio $\lambda_{i}$ is also included as an input, because the amount of computational resources to allocate may be related to how much computation is being offloaded. Another independent MLP network maps this input to the two parameters $\left(a_{\alpha },b_{\alpha }\right)$ of a Beta distribution, also processed by Softplus. The allocation ratio $\alpha_{i}$ is then sampled from the $Beat\left(a_{\alpha },b_{\alpha }\right)$ distribution.

The final output of the Actor network is the joint action $\left(K_{i},\lambda_{i},\alpha_{i}\right)$. During the training process, it is also necessary to calculate the log-probability of taking this action, $\log\pi \left(K_{i},\lambda_{i},\alpha_{i}|S_{t}\right)$, which is the sum of the log-probabilities of each independent (or conditionally independent) action part, which is calculated as follows:

(24) $$\log\pi \left(K_{i},\lambda_{i},\alpha_{i}|S_{t}\right)=\log P\left(K_{i}|S_{t}\right)+\log P\left(\lambda_{i}|S_{t},K_{i}\right)+\log P\left(\alpha_{i}|S_{t},K_{i},\lambda_{i}\right)$$

The purpose of the Critic network is to learn a state-value function $V^{\pi }\left(S_{t}\right)$, used to evaluate the expected cumulative return that can be obtained by following policy π from the current state $S_{t}$. The output of the Critic network is used to calculate the advantage function, thereby guiding the update of the Actor network. The input to the Critic network is similar to that of the Actor network, aimed at comprehensively capturing the current state information. To obtain a fixed-size state representation, we aggregate the embeddings of all RSUs, for example, by using average pooling to obtain a single RSU node embedding, and then concatenate it with the embedding of the current task’s source vehicle $h_{veh}$. The task feature vector and global statistics are the same as for the Actor network. The Critic network is a multi-layer perceptron, containing several hidden layers and one output layer. The output layer of the Critic network is a single neuron, which outputs a scalar value $V\left(S_{t}\right)$, an estimate of the value of the current state $S_{t}$.

During the training process, the state-value estimates $V\left(S_{t}\right)$ and $V\left(S_{t+1}\right)$ from the Critic network, along with the received immediate reward $r_{t}$, are used to calculate the advantage function $A\left(S_{t},a_{t}\right)$ using a method like generalized advantage estimation (GAE) [21], as follows:

(25) $${\hat{A}}_{t}^{GAE}\left(\gamma ,\lambda_{GAE}\right)=\sum_{l=0}^{\infty }{\left(\gamma \lambda_{GAE}\right)}^{l}\delta_{t+l}$$

where $\delta_{t}=\;r_{t}+\;\gamma \;V\left(S_{t+1}\right)-\;V\left(S_{t}\right)$ is the temporal-difference error, γ is the discount factor, and $\lambda_{GAE}$ is the smoothing parameter for GAE. This advantage function ${\hat{A}}_{t}^{GAE}$ measures how much better or worse performing action $a_{t}$ is compared to the average action in state $S_{t}$, and it is used to update the Actor network’s policy.

To illustrate this process, consider the following practical example: An RSU, let us call it $r_{a}$, has low computational load and high capacity. Its initial feature vector would reflect this. The GNN encoder, through its neighborhood aggregation layers, will create a final embedding $h_{a}$ for $r_{a}$. This embedding not only encodes its own favorable state but incorporates features from its neighbors. If $r_{a}$ is connected to other uncongested RSUs via low-latency R2R links, its embedding $h_{a}$ will be further refined to represent a highly accessible and powerful resource.

When a vehicle generates a compute-intensive task, the Actor network formulates a query vector $q_{att}$ that, through training, learns to represent the “ideal” target for such a task. In the high-dimensional embedding space, the model learns to place the embeddings of desirable RSUs like $h_{a}$ “closer” to such query vectors. The attention mechanism then computes the similarity (via a scaled dot-product) between $q_{att}$ and $h_{a}$. Because they are semantically aligned, they will produce a high attention score. By contrast, a heavily loaded $r_{b}$ will have an embedding $h_{b}$ that is “far” from $q_{att}$, resulting in a low score. In this way, the attention mechanism intelligently identifies $r_{a}$ as a high-quality offloading target, not just based on its own features, but on the rich, contextualized understanding of its state provided by the GNN. The entire forward pass of the Actor network, from receiving state information to generating the joint action, is summarized in Algorithm 2.
**Algorithm 2:** Actor Network Forward Pass for Joint Action Generation$\mathrm{Input}:\;\mathrm{GNN}\;\mathrm{node}\;\mathrm{embeddings}\;H,\;\mathrm{vehicle}\;\mathrm{embedding}\;h_{veh}$$,\;\mathrm{task}\;\mathrm{features}\;X_{task}$$,\;\mathrm{global}\;\mathrm{stats}\;g_{stats}$$\mathrm{Output}:\;\mathrm{Joint}\;\mathrm{action}\;\left(K_{i},\lambda_{i},\alpha_{i}\right)$$,\;\log\;\mathrm{probability}\;\log\pi \left(K_{i},\lambda_{i},\alpha_{i}|S_{t}\right)$1: **Phase A: Select Offloading Target via Attention**
$2:\;q_{att}$$\;\leftarrow \;{MLP}_{query}\left(\left[h_{veh}⊕X_{task}⊕g_{stats}\right]\right)$$3:\;C_{target}$ ← [h_veh, h_rsu_1, ..., h_rsu_M]$4:\mathrm{for}\;\mathrm{each}\;\mathrm{candidate}\;c_{j}$$\;\mathrm{in}\;C_{target}$ **do**$5:\;e_{j}$$\;\leftarrow \;(q_{att}$$\;\cdot \;c_{j}$) / $\mathrm{sqrt}\;(D_{emb}$)
6: **end for**
7: **Mask scores for invalid/unavailable targets**
$8:\;P_{target}$ ←Softmax$\left([e_{0},e_{1},\ldots ,e_{M}]\right)$$9:\;\mathrm{Sample}\;\mathrm{target}\;\mathrm{index}\;K_{i}$$\;\sim \;\mathrm{Categorical}\;(P_{target}$)
$10:\;h_{target}$$\;\leftarrow \;C_{target}$$\;[K_{i}$]
11: **Phase B: Determine Continuous Ratios**
$12:\;\mathrm{if}\;K_{i}$ = 0 **then** 
$13:\;\lambda_{i}$$\;\leftarrow \;0,\;\alpha_{i}$ ← 1
14: **else**
$15:\;a_{\lambda },b_{\lambda }$$\;\leftarrow \;Softplus\left({MLP}_{\lambda }\left(\left[h_{veh}⊕{h_{target}⊕X}_{task}⊕g_{stats}\right]\right)\right)$ + 1
$16:\;\mathrm{Sample}\;\mathrm{offload}\;\mathrm{ratio}\;\lambda_{i}$$\;\sim \;Beat\left(a_{\lambda },b_{\lambda }\right)$$17:\;a_{\alpha },b_{\alpha }$$\;\leftarrow Softplus\left({MLP}_{\alpha }\left(\left[h_{veh}⊕{h_{target}⊕X}_{task}⊕g_{stats}⊕\lambda_{i}\right]\right)\right)$ + 1
$18:\;\mathrm{Sample}\;\mathrm{resource}\;\mathrm{ratio}\;\alpha_{i}$$\;\sim \;Beat\left(a_{\alpha },b_{\alpha }\right)$19: **end if**
20: **Calculate total log probability log π from the distributions**
$21:\mathrm{return}\;\left(K_{i},\lambda_{i},\alpha_{i}\right)$$,\;\log\pi \left(K_{i},\lambda_{i},\alpha_{i}|S_{t}\right)$

### 4.4. Policy Optimization Algorithm

To effectively train the attention-based Actor–Critic policy network, the GAPO algorithm employs a deep reinforcement learning algorithm—proximal policy optimization (PPO) [19]. PPO is well-known for its excellent balance between sample efficiency, implementation simplicity, and performance stability, making it a popular choice for solving complex problems with continuous and discrete action spaces.

PPO belongs to the family of policy gradient (PG) methods which directly optimize a parameterized policy $\pi_{\theta }\left(a_{t}|S_{t}\right)$ to maximize the expected cumulative return. Compared to traditional PG methods, PPO introduces a special “clipping” mechanism or an adaptive KL-divergence penalty to constrain the step size of each policy update. This helps to avoid drastic performance drops caused by a single large update, thereby improving training stability.

In GAPO, we use the clipped surrogate objective function version of PPO. Its core idea is to ensure that the new policy does not deviate too far from the old policy during each update.

As described in Section 4.3, the Actor network outputs the parameters of the probability distribution for the joint action $\left(K_{i},\lambda_{i},\alpha_{i}\right)$. When interacting with the environment to collect data, actions are sampled from these distributions according to the current policy. The Critic network learns a state-value function, which is used to evaluate the expected cumulative return of following the current policy from state $S_{t}$.

The Actor network is updated by maximizing the following clipped surrogate objective function $L^{CLIP}\left(\theta^{\prime }\right)$ as follows:

(26) $$L^{CLIP}\left(\theta^{\prime }\right)=E_{t}\left[min\left(\frac{{\pi_{\theta^{\prime }}}_{\left(a_{t}|S_{t}\right)}}{{\pi_{\theta }}_{\left(a_{t}|S_{t}\right)}}\right){\hat{A}}_{t}^{\pi_{\theta }},clip\left(\frac{{\pi_{\theta^{\prime }}}_{\left(a_{t}|S_{t}\right)}}{{\pi_{\theta }}_{\left(a_{t}|S_{t}\right)}},1-\epsilon_{clip},1+\epsilon_{clip}\right){\hat{A}}_{t}^{\pi_{\theta^{\prime }}}\right]$$

where $\pi_{\theta^{\prime }}$ is the current policy, ${\hat{A}}_{t}^{\pi_{\theta^{\prime }}}$ is the GAE advantage estimate calculated from data collected under the old policy $\pi_{\theta }$; the $clip\left(x,low,high\right)$ function clips x into the range $\left[low,high\right]$; and $\epsilon_{clip}$ is a small hyperparameter that defines the clipping range.

This objective function limits the magnitude of the policy update by taking the minimum of two terms. If the probability ratio $\frac{{\pi_{\theta^{\prime }}}_{\left(a_{t}|S_{t}\right)}}{{\pi_{\theta }}_{\left(a_{t}|S_{t}\right)}}$ would lead to a large increase (when the advantage is positive) or a large decrease (when the advantage is negative) in the objective function, the clipping mechanism takes effect, preventing the policy from updating too quickly.

The Critic network is updated by minimizing the mean squared error (MSE) between its predicted state value and the actual return. The target return is to minimize the difference with the Monte Carlo return as follows:

(27) $$L^{VF}\left(\phi_{critic}\right)=E_{t}\left[{\left(V_{\phi_{critic}}\left(S_{t}\right)-R_{t}^{target}\right)}^{2}\right]$$

where $R_{t}^{target}$ is an estimate of the true value of state $S_{t}$.

The critical link between these mathematical updates and the physical reality of traffic load capacity is established through two core mechanisms: the reward function and the GNN-based state representation. Our multi-objective reward function is explicitly designed to penalize undesirable outcomes. When an action leads to a decision that causes high task latency or sends traffic over a congested link, the agent receives a low or negative reward. During the update process, via Equation (26), this low reward translates into a low or negative advantage estimate. Consequently, the PPO algorithm will update the Actor’s policy to reduce the probability of taking such a detrimental action in similar states in the future. The GNN encoder provides the necessary context for this learning to be effective. It processes the raw network state, including the $\rho_{link}$ for every communication link. When a link is congested, this high utilization value is encoded into the node embeddings. The Critic network, by observing these embeddings, learns to associate congested network states with lower expected future rewards, producing a more accurate value estimate. The Actor, in turn, receives these rich, congestion-aware state representations and learns a more nuanced policy that can distinguish between a high-capacity free link and a high-capacity but already saturated link. In essence, when the traffic load approaches or exceeds capacity, the environment provides a “punishment” signal via the reward. The GNN ensures the agent “sees” the congestion, and the Actor–Critic update mechanism is the process by which the agent learns from this punishment to make better, congestion-aware decisions.

The total loss function for the Actor and Critic networks is a weighted sum of their respective losses, which is calculated as follows:

(28) $$L^{TOTAL}\left(\theta^{\prime },\phi_{critic}\right)=L^{CLIP}\left(\theta^{\prime }\right)+c_{vf}\cdot L^{VF}\left(\phi_{critic}\right)$$

This total loss function is optimized via stochastic gradient descent.

Through the stable learning process of the PPO algorithm, GAPO can effectively optimize its complex policy network, which includes GNNs and attention mechanisms, thereby learning a high-performance multi-objective task offloading and resource allocation policy in a multi-hop VEC environment.

### 4.5. Convergence and Complexity Analysis

The core of the GAPO algorithm is a deep reinforcement learning framework based on proximal policy optimization (PPO). The convergence of PPO has been theoretically proven and extensively validated in practice for standard Markov decision processes (MDPs). Our analysis aims to connect these theoretical foundations to our complex multi-hop VEC environment.

The task offloading problem in vehicular edge computing can be modeled as a large-scale MDP, defined by the tuple $\left(S,A,P,R,\gamma \right)$. The state space S is represented by the dynamic graph $G_{t}$, which includes all nodes and their attributes. Due to vehicle mobility and random task arrivals, this is a high-dimensional, continuous, and dynamically changing state space. Our proposed GNN encoder aims to learn a low-dimensional and informative state representation $s_{t}=F_{GNN}\left(G_{t}\right)$, which is intended to approximate the Markov property, i.e., $P\left(s_{t+1}|s_{t},a_{t}\right)\approx P\left(s_{t+1}|s_{t},a_{t},\ldots ,s_{0},a_{0}\right)$. The action space A is a hybrid action space containing the discrete offloading destination selection $K_{i}$ and the continuous resource allocation ratios $\left(\lambda_{i},\alpha_{i}\right)$. The transition probability P is determined by the dynamics of the VEC environment. As described in Section 3.4, the reward function R is a multi-objective weighted reward function combining latency and network congestion.

Due to the high dimensionality and dynamics of the state space and the hybrid nature of the action space, traditional tabular reinforcement learning methods are not feasible. We must use function approximators. We use an Actor network with parameters θ and a Critic network with parameters ϕ to approximate the optimal policy and state-value function, respectively. Function approximation introduces errors, which is a core issue that must be addressed in convergence analysis.

Our goal is to prove that by optimizing the PPO clipped surrogate objective function, GAPO’s policy parameters θ can converge, causing the policy performance $J\left(\theta \right)=E_{\gamma \sim \pi_{\theta }}\left[\sum_{t=0}^{T}\gamma^{t}R\left(S_{t},A_{t}\right)\right]$ to reach a local optimum. To construct the proof, we assume that the GNN encoder and the Critic network have a bounded approximation error for the true value function $V^{*}\left(S_{t}\right)$. That is, there exists a small constant $\varepsilon_{V}$ as follows:

(29) $${SUP}_{S_{t}\in S}\left|V^{*}\left(S_{t}\right)-V_{\phi }\left(F_{GNN}\left(G_{t}\right)\right)\right|\leq \varepsilon_{V}$$

This assumption indicates that our GNN can effectively extract key state information. Additionally, since the reward function and value function outputs are bounded, the advantage function estimate has an upper bound $A_{max}$.

**Lemma** **1.** *For any two policies* $\pi_{\theta }$ *and* $\;\pi_{\theta^{\prime }}$*, their performance difference can be expressed as follows:*

(30) $$J\left(\theta^{\prime }\right)-J\left(\theta \right)=E_{\gamma \sim \pi_{\theta^{\prime }}}\left[\sum_{t=0}^{\infty }\gamma^{t}A^{\pi_{\theta }}\left(S_{t},a_{t}\right)\right]$$

*where* $A^{\pi_{\theta }}\left(S_{t},a_{t}\right)$ *is the true advantage function under the old policy* $\pi_{\theta }$.

Directly optimizing the expression in Lemma 1 is difficult because its expectation is based on the unknown future policy $\pi_{\theta^{\prime }}$. PPO addresses this by introducing a surrogate objective function that is a lower bound of the original objective within a neighborhood of θ′. We use the clipped surrogate objective function $L^{CLIP}\left(\theta^{\prime }\right)$ described in Section 4.4.

**Theorem** **1.** *Performing one gradient ascent update on* $L^{CLIP}\left(\theta^{\prime }\right)$ *can guarantee an approximate monotonic improvement in policy performance. Specifically, there exists a term* $\delta_{k}>0$ *, which depends on function approximation errors and sampling errors, as follows:*

(31) $$J\left(\theta_{k+1}\right)\geq J\left(\theta_{k}\right)-\delta_{k}$$

**Proof** **of** **Theorem** **1.** According to the analysis of PPO by Schulman et al. [22], a lower bound on the performance improvement can be established. □

**Theorem** **2.** *The performance sequence* ${\left\{J\left(\theta_{k}\right)\right\}}_{k=0}^{\infty }$ *corresponding to the policy sequence* ${\left\{\pi_{\theta_{k}}\right\}}_{k=0}^{\infty }$ *generated by the GAPO algorithm converges, and its policy parameter gradient converges to zero, which is calculated as follows:*

(32) $$\underset{k\to \infty }{\lim}\left\|\nabla_{\theta }J\left(\theta_{k}\right)\right\|=0$$

**Proof** **of** **Theorem** **2.** Since the reward function R has an upper bound $R_{max}$, the total expected return $J\left(\theta \right)$ also has an upper bound, as follows:

(33) $$J\left(\theta \right)\leq \sum_{t=0}^{\infty }\gamma^{t}R_{max}=\frac{R_{max}}{1-\gamma }$$

According to Theorem 1, the policy performance sequence $\left\{J\left(\theta_{k}\right)\right\}$ is approximately monotonically non-decreasing. A real-valued sequence that is approximately monotonically non-decreasing and has an upper bound must converge. Therefore, $\underset{k\to \infty }{\lim}J\left(\theta_{k}\right)$ exists. Since the performance sequence converges, the difference between its adjacent terms must approach zero. According to policy gradient theory and the PPO update mechanism, performance improvement is related to the norm of the policy gradient. When performance no longer improves, it indicates that the policy parameter updates have stabilized, i.e., the gradient $\nabla_{\theta }J\left(\theta_{k}\right)$ approaches 0. Therefore, the policy parameters of the GAPO algorithm will converge to a stationary point, which corresponds to a locally optimal task offloading and resource allocation policy.

Regarding the computational complexity of the GAPO algorithm during a single offloading decision, we mainly focus on the inference phase complexity, as it directly affects the system’s real-time response capability. Let the system have N vehicles and M RSUs, so the total number of nodes is $N_{t}=\;N+M$, and the total number of edges is $E_{t}$. The GNN embedding dimension is $D_{emb}$. The complexity of the GNN is primarily determined by message passing. For one GAT layer, its complexity is $O\left(N_{t}D_{in}D_{out}+E_{t}D_{out}\right)$. For an L-layer GNN, the total complexity is approximately $O\left({L\cdot E}_{t}{\cdot D}_{emb}\right)$, as the number of edges is typically much larger than the number of nodes. The query vector $q_{att}$ in the Actor network is generated by a small MLP, with a complexity that can be considered $O\left(1\right)$. The attention score calculation involves the dot product of the query vector with M + 1 candidate target embeddings, with a complexity of $O\left(M{\cdot D}_{emb}\right)$. The two continuous actions are generated by two small MLPs, with complexity $O\left(1\right)$. In summary, the total complexity of a single decision is dominated by the GNN encoder, which is $O\left(N_{t}D_{in}D_{out}+E_{t}D_{out}\right)$. Since the network topology is sparse, $E_{t}$ has a linear relationship with $N_{t}$. Therefore, the complexity can be approximated as $O\left(N_{t}{\cdot D}_{emb}\right)$. The computational complexity of GAPO is polynomial with respect to the network size, making it practically applicable in dynamic VEC environments. □

## 5. Experimental Analysis

To systematically evaluate the performance of the proposed GAPO algorithm in dynamic multi-hop VEC environments, this chapter designs a series of comparative and ablation experiments. We first introduce the experimental environment, parameter configurations, and training hyperparameters. Then, we provide a detailed description of the baseline algorithms used for comparison. Finally, by presenting and analyzing simulation result charts, we conduct an in-depth analysis of the performance of GAPO and other algorithms under different scenarios, verifying its superiority in minimizing task latency and alleviating R2R link congestion.

### 5.1. Experimental Setup

We conduct our experiments in a simulated unidirectional multi-lane highway scenario. This scenario consists of a series of dynamically moving vehicles and fixedly deployed roadside units. Vehicles randomly generate computational tasks and make decisions to either process them locally or offload them to one or more RSUs. The key parameters of the simulation environment are listed in Table 2. These parameters constitute the baseline scenario for evaluating the performance of our algorithm. The key hyperparameters for the training process are shown in Table 3.

The selection of key hyperparameters is critical to the performance and stability of the GAPO algorithm. Below, we provide the rationale for our choices, which are based on the established literature and empirical observations from our experiments. The output dimension of the GNN encoder determines the richness of the learned node representations. A dimension of 32 was chosen to strike a balance between model expressiveness and computational cost. It is large enough to capture complex node features and topological context but small enough to prevent overfitting and to ensure that the GNN encoder can operate efficiently for timely decision-making. Our ablation studies (Section 5.3) show that this dimension, when combined with the full model, yields excellent performance, confirming its adequacy. Following the original GATv2 architecture [20], we employ a multi-head attention mechanism. Using multiple heads allows the model to learn different weighted aggregations of neighbor information in parallel, capturing more diverse relationship patterns and stabilizing the learning process. This is particularly useful in our heterogeneous VEC environment, where the importance of a neighbor may depend on various factors. The learning rate is a crucial parameter for training stability. We selected a relatively conservative value of 1 × 10^-4^, which is a common and robust choice for PPO with the Adam optimizer. This smaller learning rate helps prevent destructive policy updates, especially given the complexity of our model and the dynamic nature of the environment. The stable convergence shown in our training curves validates that this rate facilitates effective learning without policy collapse.

### 5.2. Comparative Experiments

To comprehensively evaluate the performance of GAPO, we selected several representative baseline algorithms for comparison, covering traditional strategies, heuristic methods, and other reinforcement learning approaches.

- Local-Only: All tasks generated by vehicles are processed on their local on-board units, without any network offloading. This strategy completely avoids network communication latency and congestion but is limited by the finite computational power of the vehicles.
- Random Offload: A simple baseline strategy where vehicles make decisions randomly. It randomly selects an available RSU as the offloading target or chooses to process the task locally. If offloaded, the task is fully offloaded.
- Greedy Lowest Load: A heuristic algorithm where, at the time of decision, the vehicle evaluates the expected computational load of all reachable computation nodes, including itself. It always greedily selects the node with the lowest computational load as the offloading target.
- A2C (Advantage Actor–Critic) [23]: A classic reinforcement learning algorithm. To adapt it to the complex environment of this study, we designed a manual feature engineering module for it. This module transforms the graph-structured observation into a fixed-length vector. Specifically, it extracts a series of aggregated features for the current task, the source vehicle, and each RSU, such as direct connectivity with the source vehicle, the number of hops and estimated latency of the multi-hop path to the RSU, and the RSU’s own computational load. These features are concatenated into a long vector and fed into a standard MLP-based Actor–Critic network. The performance of the A2C algorithm is highly dependent on the completeness and effectiveness of these hand-crafted features, which stands in stark contrast to GAPO’s core idea of automatically learning network state representations via GNN.

To evaluate the performance of GAPO, we designed five different scenarios focusing on three core dimensions: network scalability, resource and load adaptability, and environmental heterogeneity. We tested each algorithm for 50 episodes in each scenario. We primarily focus on two key metrics: Average Task Latency and Average R2R Link Congestion. The latter measures the degree of congestion on the R2R backbone links caused by offloading traffic, calculated as the average of the actual utilization exceeding the stability threshold φ across all R2R links involved in task transmissions.

**Figure 5:**
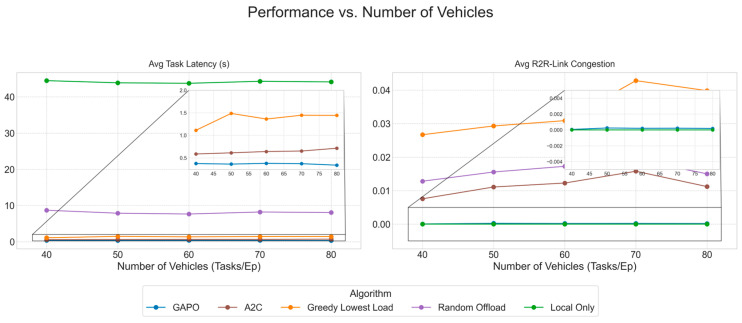
Impact of Vehicle Count on Performance.

Given that the task offloading problem is NP-hard, assessing the scalability of any proposed algorithm is of paramount importance. As the problem size grows, the solution space expands exponentially, making it intractable for exact methods and posing a significant challenge for heuristics and learning-based approaches. Figure 5 directly evaluates the scalability of all tested algorithms by increasing the number of vehicles from 40 to 80, which proportionally increases the system load and resource competition. The results reveal a clear distinction in scalability. The performance of both the greedy lowest load and the A2C algorithms deteriorates significantly as the network scale increases. The greedy algorithm’s myopic nature becomes its downfall at scale; with more vehicles making decisions, the probability of creating traffic “hotspots” at the computationally strongest RSUs increases, leading to disproportionately severe R2R link congestion. Similarly, the classic A2C algorithm, which relies on a flattened, fixed-size state vector, suffers from an information bottleneck. As the network grows, this vector becomes increasingly inadequate at capturing the complex, expanding web of topological relationships, resulting in a sharp decline in decision quality. By contrast, GAPO demonstrates superior scalability, a direct result of its architectural design. The core of this advantage lies in the GNN encoder. GNNs process information locally through message passing, meaning the computation for a node’s embedding depends on the size of its immediate neighborhood, not the total number of nodes in the graph. This inherent locality prevents the “curse of dimensionality” that plagues the A2C model. Consequently, as the network scales, GAPO can efficiently and effectively continue to generate high-quality, context-aware state representations. This allows it to maintain low latency and minimal congestion, proving its viability as a practical solution for large-scale instances of this NP-hard problem.

**Figure 6:**
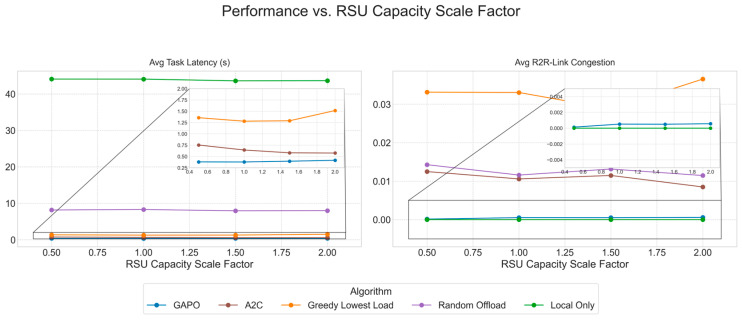
Impact of RSU Computational Capacity on Performance.

We evaluated resource adaptability by adjusting the computational capacity of all RSUs with a scaling factor. As shown in Figure 6, when the RSU computational capacity increases, the latency of all offloading algorithms decreases. GAPO leads significantly with the lowest latency and R2R congestion. It is worth noting that, even when RSU capacity is low (factor of 0.5), GAPO still maintains extremely low latency, demonstrating its ability to efficiently utilize limited computational resources. Although the runner-up algorithm, A2C, also benefits from the capacity increase, its performance improvement is not as significant as GAPO’s, and it is consistently accompanied by higher R2R congestion, indicating its failure to effectively co-optimize computation and communication resources.

**Figure 7:**
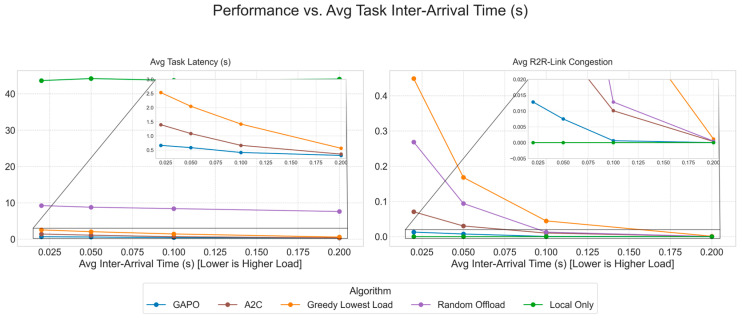
Impact of Task Arrival Interval on Performance.

We tested system load adaptability by varying the task Inter-Arrival Time (IAT). In Figure 7, when the system enters the high-load, extreme-pressure scenario (IAT = 0.02 s), the performance gap between the algorithms widens dramatically. GAPO still maintains the best performance, with its latency and R2R congestion increasing much less than the other algorithms. This highlights GAPO’s adaptability in highly dynamic and competitive environments. Its core advantage lies in the fact that, when the entire network becomes congested, the global contextual information provided by the GNN becomes crucial. By contrast, A2C’s fixed feature combination can no longer effectively represent the complex network state under high load, leading to a drop in decision quality. GAPO is able to discover non-greedy but globally superior offloading paths, such as choosing a slightly closer but slightly higher-load RSU to avoid the backbone link paralysis caused by excessive traffic concentration on a few optimal RSUs.

**Figure 8:**
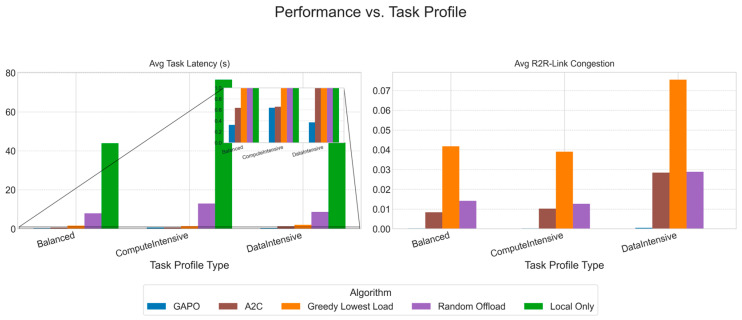
Impact of Task Type Heterogeneity on Performance.

Real-world VEC environments are often heterogeneous. We set up two scenarios, task heterogeneity and RSU computational resource heterogeneity, to evaluate the algorithm’s performance under environmental heterogeneity. In the task heterogeneity scenario, we tested three task types: balanced, compute-intensive, and data-intensive. The balanced type uses the default task data and computation sizes. The compute-intensive type maintains the default data size but increases the computation size to (1.0, 2.5) Gc. The data-intensive type maintains the default computation size but increases the data size to (800, 1024) KB. The experimental results are shown in Figure 8. For compute-intensive tasks, the latency of Local Only skyrockets to about 76 s, highlighting the bottleneck of the vehicle’s local computational capacity. GAPO still performs optimally, proving its ability to intelligently match heavy computation tasks to the most suitable RSU resources. For data-intensive tasks, which require large amounts of data transmission, the demands on network bandwidth are higher. In this case, A2C’s R2R link congestion rate increases significantly compared to the balanced type, indicating that classic reinforcement learning algorithms struggle to fully capture the global network topology and inter-node dependencies.

**Figure 9:**
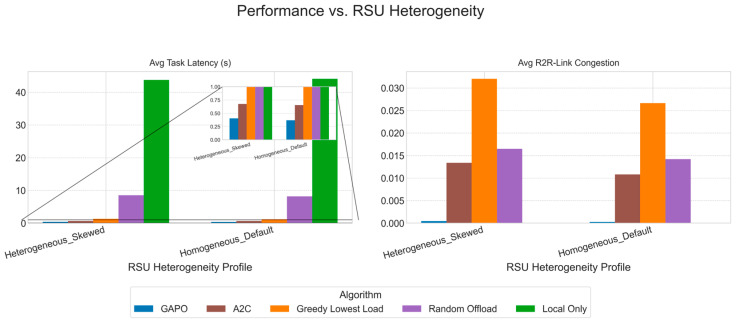
Impact of RSU Computational Resource Heterogeneity on Performance.

To evaluate the impact of RSU computational resource heterogeneity, we set up “Skewed” and “Default” heterogeneity modes. In the “Skewed” mode, the RSU compute capacities are set to [10.0, 10.0, 15.0, 30.0, 40.0] GHz. In the “Default” mode, they are [15.0, 20.0, 25.0, 30.0, 35.0] GHz. As shown in Figure 9, in the “Skewed” scenario, a few RSUs possess far superior computational power than others. This imbalanced resource distribution poses a severe challenge to load balancing strategies. The experimental results show that greedy lowest load and A2C perform poorly in this scenario, leading to high R2R congestion. They tend to offload a large number of tasks to the few “super” RSUs, causing severe congestion on the paths to these RSUs and creating a “hotspot” effect. By contrast, by leveraging its awareness of global topology and load, GAPO successfully avoids this trap. It understands that diverting traffic to some slightly weaker but more accessible RSUs can achieve lower overall latency and better system stability, thus realizing true global load balancing.

The substantial performance disparities observed across the algorithms in Figure 5, Figure 6 and Figure 7 are not arbitrary; they directly reflect the fundamental differences in their underlying logic and awareness of the network state. The Local Only strategy, by design, serves as a stark baseline, consistently showing the highest latency because it completely forgoes the benefits of edge computing. It forces all tasks onto the vehicle’s computationally weak on-board unit, creating an immediate and severe processing bottleneck that underscores the critical need for an effective offloading mechanism. The greedy lowest load algorithm represents a step toward utilizing edge resources, yet its performance is severely hampered by a myopic and communication-blind approach. Its sole focus on instantaneous computational load causes it to overlook the substantial communication latency required to reach a distant, albeit computationally less loaded, RSU. This flawed logic not only incurs high transmission delays but systematically creates network hotspots. As multiple vehicles greedily select the same “optimal” server, they funnel traffic onto the same backbone paths, leading to the severe R2R link congestion consistently observed in our results. The algorithm’s attempt to find the least loaded server paradoxically leads to systemic congestion and high end-to-end latency. In stark contrast, GAPO’s superior performance is a direct result of its ability to achieve a holistic, globally-aware optimization. Through its GNN encoder, GAPO builds a rich, contextualized representation of the entire network graph, where each node’s embedding reflects not only its own load but the congestion state of its connecting links and neighbors. Armed with this global perspective, the DRL agent, guided by a multi-objective reward function, learns a far more sophisticated policy than a simple greedy choice. It learns to navigate the inherent trade-off between computation and communication, understanding that offloading to a slightly busier but closer RSU is often vastly superior to choosing a distant, idle server over a congested path. By effectively co-optimizing both latency and congestion, GAPO avoids the creation of hotspots and consistently achieves the lowest true end-to-end task completion time, thus explaining its significant and robust advantage across all tested scenarios.

In summary, the comprehensive comparison across multiple dimensions consistently demonstrates the significant superiority of the GAPO algorithm. Its core advantage lies in the powerful network state representation capability endowed by the GNN and the intelligent decision-making ability driven by the attention mechanism. This enables GAPO to co-optimize the two key objectives of latency and congestion in complex, dynamic multi-hop VEC environments, providing an efficient and robust task offloading solution that surpasses traditional methods and other DRL algorithms.

### 5.3. Ablation Study

To further validate the effectiveness of the graph neural network encoder and the attention-based Actor network in the GAPO algorithm, we conducted a series of ablation studies. By comparing with two key simplified variants, we can quantify the contribution of each component to the overall performance. We designed the following two ablation variants and compared them with the full GAPO model:

1. GAPO: The complete algorithm proposed in this paper, using both the GNN encoder and the query-based attention mechanism for target selection.
2. noAtt: This variant removes the query-based attention module from the Actor network. Instead, it directly concatenates the source vehicle’s embedding, each candidate target’s embedding, and the task features, then uses a simple MLP network to score each candidate, finally selecting a target via Softmax. This variant still uses the GNN encoder to obtain node embeddings but loses the ability to intelligently match task needs with target characteristics.
3. noGNN: This variant replaces the GNN encoder with a standard multi-layer perceptron (MLP). In this model, the features of each node are input into the MLP independently, completely ignoring the network topology. Therefore, this model cannot learn the contextual information of nodes through neighbor aggregation, and its state-aware capability degenerates to be similar to traditional flattened state vector methods.

We trained and tested these three models in the baseline scenario. The final performance metrics are summarized in Table 4.

The data in Table 4 leads to a clear conclusion. The noGNN variant performs the worst, with its average latency being 82.1% higher than the full GAPO model, and its R2R link congestion being nearly 30 times higher. This is not merely a quantitative difference but reveals a fundamental flaw. The root cause lies in its inability to perceive network topology. The noGNN variant, which uses a standard MLP, processes each node’s feature vector in isolation. It can see the attributes of individual RSUs but is completely blind to the state of the communication links connecting them.

Consequently, the noGNN agent cannot grasp the cumulative effect of multi-hop link loads. It might identify an RSU that is computationally powerful and has low local load, but it is unaware that the two- or three-hop path to reach it is severely congested. This leads the agent to make myopic, greedy decisions, repeatedly attempting to offload tasks to the most powerful RSUs on paper. This behavior creates severe network “hotspots”, funneling traffic onto a few backbone links and causing the massive R2R congestion we observe. This result strongly proves that the global network topology and contextual awareness provided by the GNN are the cornerstone of achieving efficient task offloading.

The performance of the noAtt variant lies between the other two, with its average latency being 27.5% higher than the full GAPO model. This indicates that, even with the high-quality state representation provided by the GNN, a simple and coarse decision-making mechanism (concatenation and MLP scoring) cannot fully utilize this information. The query-based attention mechanism is crucial for achieving more fine-grained target selection by dynamically matching task requirements with candidate target characteristics, thereby further optimizing latency and congestion.

**Figure 10:**
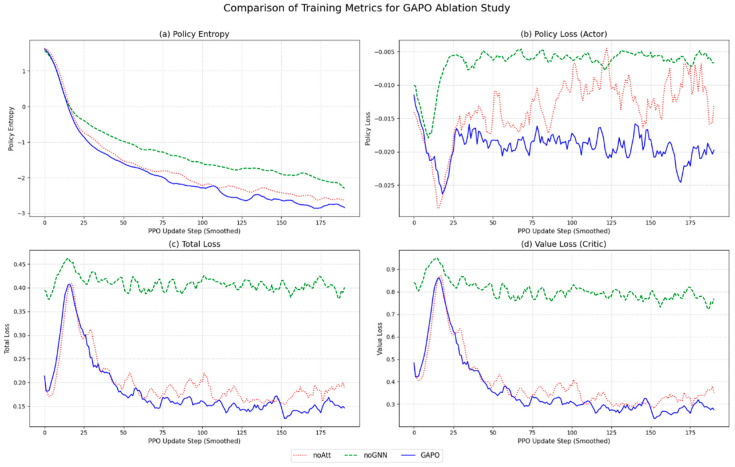
Ablation Model Training Metrics. (**a**) Policy Entropy. (**b**) Actor Network Policy Loss. (**c**) Total Loss. (**d**) Critic Network Policy Loss.

To further investigate the impact of these components on the learning process, we plotted and analyzed the key metrics during model training, as shown in Figure 10. Subplot (a) shows the trend of policy entropy. Entropy measures the degree of exploration or uncertainty in the policy. The entropy of all models decreases during training, indicating that the agents are gradually transitioning from exploration to exploitation, learning more deterministic policies. Among them, the entropy of noGNN decreases the slowest and has the highest final value, indicating that it had difficulty finding an effective policy and consistently maintained a high level of uncertainty. Subplots (b) and (d) show the Actor and Critic losses, respectively. The policy loss and value loss of noGNN are not only the highest in value but are extremely volatile. This reveals a deeper problem: because the noGNN variant cannot accurately perceive the network state, its Critic network fails to produce reliable state-value estimates. This results in noisy advantage signals, which in turn makes the Actor’s policy updates difficult and unstable. By contrast, the loss curves of GAPO and noAtt are lower and more stable, demonstrating that the state representation provided by the GNN greatly improves the stability and efficiency of learning. Among them, the full GAPO model has the lowest loss, indicating that the attention mechanism also plays a positive role in stabilizing the learning process.

In summary, the comprehensive comparison across multiple dimensions consistently demonstrates the significant superiority of the GAPO algorithm. The experimental results show that GAPO consistently outperforms traditional and heuristic-based algorithms. Its core advantage lies in the synergy between the GNN encoder and the DRL policy network. While GNN-informed heuristic methods, such as those relying on shortest-path routing over predicted link weights [24], can improve upon context-agnostic approaches, our DRL-based policy is capable of learning more complex and robust strategies. It can dynamically balance immediate costs with potential future congestion, achieving a superior trade-off between task latency and network stability.

## 6. Discussion

Our current experimental evaluation is based on a multi-hop highway scenario, which models a linear network topology. A natural and important question is how the proposed GAPO framework would generalize to more complex and realistic urban scenarios, such as multi-intersection grids.

The core design of GAPO is fundamentally based on a graph representation of the network, which makes it inherently suitable for more complex topologies. Unlike methods tailored for linear arrangements, our use of GNNs is topology-agnostic and can naturally process arbitrary graph structures, such as the mesh-like connectivity found in urban grids. However, transitioning to such a scenario would introduce several challenges and require specific, yet feasible, adaptations.

1. State Representation and Scalability: An urban grid would contain a larger number of RSU nodes and a more complex edge set. This increases the scale of the input graph. GAPO’s GNN architecture is well-equipped to handle this. By design, GNNs like GATv2 operate on local neighborhoods. The computational complexity for updating a single node’s embedding scales with its number of neighbors, not the total number of nodes in the graph. This inherent locality ensures that the state representation module of GAPO remains scalable even in larger, denser network environments.
2. Multi-Hop Pathfinding: The pathfinding algorithm in our current implementation implicitly assumes a linear chain of RSUs. For an urban grid, this module would need to be replaced with a more general graph traversal algorithm. This is a straightforward modification. The RSU network can be treated as a subgraph, where the edges are dynamically weighted by the real-time communication latency calculated by our model. A standard algorithm like Dijkstra’s could then be directly applied to find the latency-optimal path between any two RSU nodes. This would allow GAPO to identify the most efficient multi-hop communication routes through the urban mesh network without changing the core RL decision-making logic.
3. V2V Communication at Intersections: Urban intersections present rich opportunities for multi-hop V2V relaying, which is not a primary focus in our highway model. Extending GAPO to fully leverage this would be a promising direction for future research. This could involve dynamically including high-value vehicle nodes into the pathfinding subgraph, further enriching the offloading options.

In conclusion, while a full empirical evaluation on an urban grid is a direction for future work, we argue that the fundamental design of GAPO is robust and generalizable. The necessary adaptations for handling complex topologies are well-defined and rely on standard algorithmic components, demonstrating a clear and feasible path for applying GAPO to more complex and realistic vehicular networks.

## 7. Conclusions

This paper addresses a series of challenges in task offloading decision-making within dynamic multi-hop Vehicular Edge Computing networks, including the difficulty of real-time network state perception and the complexity of the decision space. We have proposed an intelligent task offloading algorithm based on a graph attention policy optimization framework—GAPO. The core innovation of this algorithm lies in the deep integration of the powerful state-aware capabilities of graph neural networks with the sequential decision-making abilities of deep reinforcement learning. By modeling the VEC network as a dynamic attributed graph, the GNN is able to extract global, context-rich feature embeddings from complex topological structures and node states. Building on this foundation, an Actor–Critic framework based on an attention mechanism can intelligently select the optimal offloading destination according to the specific needs of the current task and jointly determine the offloading ratio and computational resource allocation. Finally, a multi-objective reward function guides the agent to learn a near-optimal policy that balances various performance indicators. Our research has designed and implemented an efficient, adaptive, and distributed task offloading framework, GAPO, providing an effective solution to the resource management problems in complex and dynamic VEC environments. While the comprehensive simulation results robustly demonstrate the effectiveness of GAPO, we acknowledge the limitations inherent in a simulation-based study. The complexities of real-world wireless channels, unpredictable vehicle mobility patterns, and diverse application workloads can introduce challenges not fully captured by even high-fidelity models. Therefore, a crucial direction for future work is to validate the performance of GAPO in a real-world hardware testbed or a large-scale, trace-driven emulation environment. Such an endeavor would not only confirm the practical viability of our approach but provide valuable insights for bridging the gap between algorithmic innovation and real-world deployment.

## Figures and Tables

**Table 1:** Summary of Key Notations.

Symbol	Description
V	Set of all vehicles in the system.
R	Set of all roadside units.
$G_{t}$	The attributed graph representing the network state at time t.
$N_{t}$	Set of all nodes at time t.
$E_{t}$	Set of all communication links at time t.
$X_{t},E_{t}$	Node and edge attribute matrices.
$T_{i}$	The i-th computational task.
$d_{i}$	Data size of task $T_{i}$(in KB).
$c_{i}$	Computational complexity of task $T_{i}$ (in giga-cycles).
$A_{i}$	The joint action taken for task $T_{i}$.
$K_{i}$	Discrete offloading destination for task $T_{i}$.
$\lambda_{i}$	Continuous offloading ratio for task $T_{i}$.
$\alpha_{i}$	Continuous computational resource allocation ratio for task $T_{i}$.
$L_{local}$	Latency for local computation.
$L_{edge}$	Latency for computation on an edge server.
$L_{v2r}$, $L_{r2r}$	Communication latency over a V2R or R2R link.
$L_{i}^{total}$	Total end-to-end completion latency for a task.
$L_{avg}$, $C_{cong}$	Average task latency and average R2R link congestion.
$J\left(X\right)$	The multi-objective function to be minimized.

**Table 2:** Simulation Environment Parameters.

Parameter	Description	Value
Highway Length	Total length of the highway in the simulation	1000 m
RSU Coverage Radius	Signal coverage range of each RSU	200 m
Default RSU Count	Number of RSUs in the baseline scenario	5
RSU Default Compute Capacity	Set of RSU compute capacities (heterogeneous)	{15, 20, 25, 30, 35} Gcps
Vehicle Local Compute Capacity	On-board compute capacity of a single vehicle	0.25 Gcps
RSU–R2R Link Base Bandwidth	Backbone link bandwidth between RSUs	30 Mbps
Default Task Arrival Interval	Average time interval between task generations	0.1 s
Task Data Size	Range of data size to be transmitted for tasks	(512,1024) KB
Task Computation Size	Range of computation cycles required for tasks	(0.5,1.5) Gc
G/G/1 Stability Factor	Factor used to calculate the effective service rate	0.99
State Averaging Window	Time window for EWMA calculation	1 s

**Table 3:** GAPO Training Hyperparameters.

Hyperparameter	Value
Learning Rate	1 × 10^−4^
Discount Factor (γ)	0.99
GAE Smoothing Parameter (λ)	0.95
Training Epochs per Update Cycle	10
Experience Replay Buffer Size	2048
Mini-batch Size for Updates	4
Entropy Loss Coefficient	0.01
Value Function Loss Coefficient	0.5
GNN Output Node Embedding Dimension	32
GAT Attention Head Count	2
MLP Hidden Layer Dimension in the Actor Network	64
MLP Hidden Layer Dimension in the Critic Network	64

**Table 4:** Ablation Study Results.

Model	Average Task Latency (s)	Average R2R Link Congestion Rate
GAPO	**0.374 (±0.178)**	**0.05%**
noAtt	0.477 (±0.197)	0.09%
noGNN	0.681 (±0.264)	1.64%

## Data Availability

The datasets presented in this article are not readily available because the data are part of an ongoing study. Requests to access the datasets should be directed to corresponding author.
